# A multidisciplinary approach to the identification of the protein–RNA connectome in double-stranded RNA virus capsids

**DOI:** 10.1093/nar/gkad274

**Published:** 2023-04-18

**Authors:** Po-yu Sung, Yiyang Zhou, C Cheng Kao, Ali A Aburigh, Andrew Routh, Polly Roy

**Affiliations:** Department of Infection Biology, London School of Hygiene and Tropical Medicine, London, UK; Department of Biochemistry and Molecular Biology, University of Texas Medical Branch, Galveston, TX, USA; Previously in the Department of Molecular & Cellular Biochemistry, Indiana University, Bloomington, IN, USA; Department of Infection Biology, London School of Hygiene and Tropical Medicine, London, UK; Department of Biochemistry and Molecular Biology, University of Texas Medical Branch, Galveston, TX, USA; Sealy Center for Structural Biology and Molecular Biophysics, The University of Texas Medical Branch, Galveston, TX, USA; Institute for Human Infections and Immunity, University of Texas Medical Branch, Galveston, TX, USA; Department of Infection Biology, London School of Hygiene and Tropical Medicine, London, UK

## Abstract

How multi-segmented double-stranded RNA (dsRNA) viruses correctly incorporate their genomes into their capsids remains unclear for many viruses, including Bluetongue virus (BTV), a *Reoviridae* member, with a genome of 10 segments. To address this, we used an RNA-cross-linking and peptide-fingerprinting assay (RCAP) to identify RNA binding sites of the inner capsid protein VP3, the viral polymerase VP1 and the capping enzyme VP4. Using a combination of mutagenesis, reverse genetics, recombinant proteins and *in vitro* assembly, we validated the importance of these regions in virus infectivity. Further, to identify which RNA segments and sequences interact with these proteins, we used viral photo-activatable ribonucleoside crosslinking (vPAR-CL) which revealed that the larger RNA segments (S1-S4) and the smallest segment (S10) have more interactions with viral proteins than the other smaller segments. Additionally, using a sequence enrichment analysis we identified an RNA motif of nine bases that is shared by the larger segments. The importance of this motif for virus replication was confirmed by mutagenesis followed by virus recovery. We further demonstrated that these approaches could be applied to a related *Reoviridae* member, rotavirus (RV), which has human epidemic impact, offering the possibility of novel intervention strategies for a human pathogen.

## Graphical Abstract

**Graphical Abstract:**
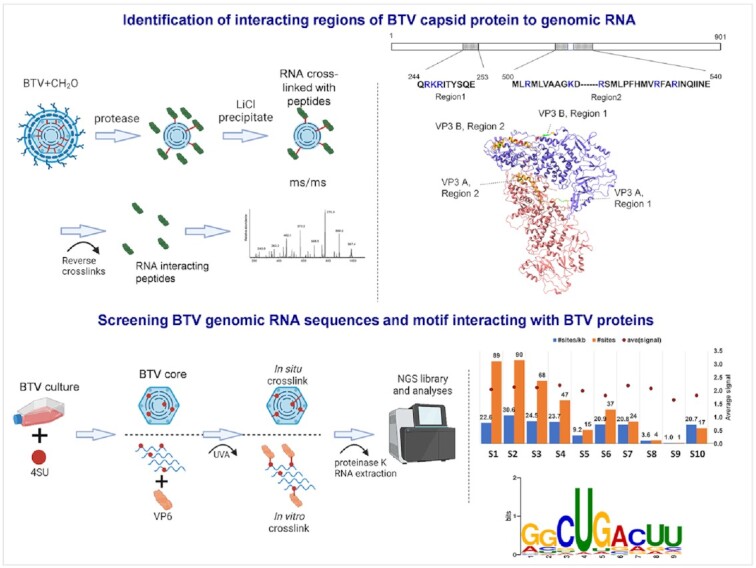
The molecular connectome of double-stranded RNA viruses.

## INTRODUCTION

How viruses correctly package their genomes represents one of the most challenging questions in virology. Genome packaging is a key process in the virus life cycle and must be highly specific, such that, a single genome or a set of genomic segments is correctly packaged into the confined space of the capsid and all other extraneous nucleic acids are excluded. Some viruses use a binding site in the viral capsid protein to recognize a specific sequence of the genome (1–3), but this mechanism becomes exceptionally challenging for viruses with multi-segmented genomes, as only a single copy of each segment must be incorporated. In the case of segmented single-stranded RNA (ssRNA) viruses, such as Cowpea Chlorotic Mottle Virus (CCMV), the length of the RNA segments is key to successful packaging (4). Whereas for others, such as Satellite Tobacco Necrotic Virus (STNV), a specific interaction between the genomic RNA and the viral capsid is vital for RNA packaging (1). Similarly, a specific interaction between genomic RNA and capsid was demonstrated to play an important role in RNA packaging of the bacteriophage φ6, which has a 3-segmented double-stranded RNA (dsRNA) genome (5).

Since the *Reoviridae* family has 10–12 genomic dsRNAs, all of which must be packaged correctly to generate a viable virus particle, it is therefore unlikely that this family of viruses had adopted a CCMV-like length-dependent packaging strategy. Instead, a specific recognition between genome and capsid seems more likely. This is supported by the fact that, following virus entry, individual ssRNA transcripts leave the icosahedral capsid by separate channels, implying an ordered network of genomic segments primed for transcription (6). Indeed, cryo-electron microscopy (Cryo-EM) has visualised the dsRNA genome segments wrapped in an apparently specific manner (7).

Bluetongue virus (BTV), a member of Orbivirus genus of *Reoviridae* family, has particles that are organized into two capsids: an outer capsid composed of two proteins (VP2 and VP5) and an inner icosahedral capsid (core) composed of two additional proteins, VP7 and VP3. Within the core, the virus encapsidates 10 dsRNA genome segments (S1 to S10) and two replication related proteins, the polymerase (VP1) and the capping enzyme (VP4). These two core proteins are common across the *Reoviridae*, but BTV and other members of the Orbivirus genus has an additional protein, VP6, that is essential for RNA packaging (8,9).

When BTV enters the cell, the outer capsid is removed releasing the inner capsid core into the cytoplasm. Without further disassembly, the released core particle synthesizes and extrudes ssRNA transcripts into the cytoplasm. While some ssRNA transcripts act as templates for viral protein synthesis, others form RNA complexes through RNA-RNA interactions and are packaged, possibly with the help of viral protein(s), into the assembling progeny capsids (10,11). Genomic dsRNA segments are then synthesized within the capsid.

Recent data suggests that a direct interaction between VP6 and VP3 is essential for viral genome packaging and the early stage of virus particle formation (12). Furthermore, evidence shows that VP6 interacts with viral RNA through selective binding sites to assist RNA packaging (9). However, whether the capsid protein VP3 also interacts with and/or recruits viral RNA has not been shown. For example, ssRNAs could be chaperoned and anchored in the viral core by VP6 alone or by direct interaction with VP3, or by a combination of these interactions. Since other members of the *Reoviridae* (e.g. rotavirus, RV) share similar assembly processes but do not possess a packaging protein analogous to VP6, understanding any additional interactions between BTV VP3 and RNA may reveal the role of capsid proteins in the RNA packaging of other *Reoviridae* members.

Most recently, newer techniques such as viral Photo-Activatable Ribonucleoside Cross-linking (vPAR-CL) have shown that it is possible to identify the specific nucleotide sequences that interact with viral proteins (13). Here, we apply this method to investigate two large and complex viruses, BTV and RV, to determine the interactions of each viral genomic RNA with the candidate viral proteins. For each virus, our data define the interactions between RNA genomic segments and the virus capsid and indicates the putative localisation of genomic RNAs within the capsid.

## MATERIALS AND METHODS

### Virus, plasmids, mutagenesis, and RNA transcript synthesis

BTV-1 (GenBank accession numbers FJ969719 to FJ969728) and RV SA11 (GenBank accession numbers LC333802 to LC333812) were used for RCAP and vPAR-CL studies. All mutations for reverse genetics and recombinant protein production were generated by site-directed mutagenesis. The sequences of the primers are listed in Supplementary Table S1. Transcripts for reverse genetic analyses were prepared using the mMACHINE T7 transcription kit (Thermo), following the manufacturers’ instructions.

### BTV cores and RV DLPs purification

The purification methods for BTV cores and RV DLPs were described previously (14,15). In brief, BTV and RV were grown in BSR cell line and MA104 cell line respectively. After 90–100% CPE is reached, the cells were harvested and the cell lysates were subjected to a 40% sucrose cushion and sequentially a continuous CsCl density gradient. CsCl was then removed from the fractions containing viral particles using ultracentrifugation.

### RCAP

The RCAP assay was carried out as described previously (9,16). Formaldehyde was added into purified BTV-1 cores and RV SA11 DLPs to a final concentration of 0.1%, and the mixture was incubated for 10 min at room temperature. Glycine was added to a final concentration of 0.2 M for 10 min to quench additional cross-linking. The cross-linked protein–RNA complexes were digested using sequencing-grade trypsin (Trypsin Gold; Promega) for 16 h at a 1:20 (wt/wt) ratio of trypsin to capsids. RNA-peptide complexes were then selectively precipitated using a final concentration of 3 M lithium chloride and centrifugation at 16 000 × g. The peptide-RNA conjugates were reversed by a 1 h incubation at 70°C. Parallel control reactions to assess background signals were performed without the addition of formaldehyde. RCAP peptides were analysed using an Orbitrap Elite hybrid ion trap mass spectrometer equipped with an electrospray ionization source (Thermo Fisher Scientific). The peptides were resolved using a Dionex UltiMate 3000 high-performance liquid chromatograph (HPLC) with a 1- by 150-mm Zorbax 300SB C18 column (Agilent) and were eluted using a linear gradient of 2–45% acetonitrile in water with 0.1% formic acid over 90 min with a flow rate of 50 μl/min. Tandem mass spectra were obtained using collision-induced dissociation in a data-dependent manner. Spectra were searched against a database of BTV-1 and RV SA11 proteins. Unspecific enzyme cleavage and a mass tolerance of 10 ppm were used.

### Generation of recombinant baculoviruses and analysis of VP3 mutants

BTV-1 segment 3 that encodes VP3 was cloned into pFastBac expression vector (ThermoFisher Inc.) and the recombinant baculoviruses were generated and selected based on manufacturer's instruction. The VP3 expression was analysed as previously described (17). Polyacrylamide gel electrophoresis (PAGE) and Western blot analysis of proteins was performed following standard protocol.

### rVP6 expression and purification

Recombinant VP6 (rVP6) of BTV-10, which is fully exchangeable with BTV-1 VP6 was used in vPAR-CL. The expression and purification of recombinant baculovirus expressing VP6 in the Sf9 cells have been described previously (18). The His-tagged reVP6 proteins were purified using Ni-NTA affinity purification following the standard protocol.

### Purification of core-like particles

Baculovirus-expressed CLPs were purified as described previously (17,19). Briefly, Sf9 cells were coinfected with recombinant baculovirus in suspension culture expressing wild-type VP7, together with either wild-type VP3 or mutant VP3, using an MOI of 5. 48 h post infection (p.i.), infected cells were harvested and lysed by Dounce homogenization in TNN buffer (20 mM Tris–HCl, pH 8, 150 mm NaCl, and 0.5% Nonidet *P*-40). Assembled CLPs were purified from the soluble fraction by centrifugation on a 35% CsCl gradient as described previously (20). The presence of CLP proteins was analysed by SDS–10% PAGE, followed by staining with Coomassie Blue.

### Reverse genetics

Mutations in the cDNA of S9 RNA that encodes VP6 were generated using site-directed mutagenesis (sequences available upon request), together with the other 9 BTV genome segments that were used to transfect BSR cells or a BSR cell line that stably expresses VP6 (BSR-VP6), as described by Boyce *et al.* (21). Cytopathic effect (CPE) was monitored after 3 days, and the mutations in S9 in the recovered viruses were confirmed by RT-PCR and sequencing.

### Plaque assay

WT and mutant viruses were diluted, applied to BSR cell monolayers at a multiplicity of infection (MOI) of 0.01–0.1, and covered by an Avicel overlay as described by Matrosovich *et al.* (22). The cells were fixed with formaldehyde and the plaque size monitored after 3 days.

### *In vitro* CFA assay

The BTV *in vitro* cell-free assembly (CFA) assay has been described previously (11). Briefly, the 10 segments of ssRNA were incubated with VP1, VP4 and VP6 for 30 min, followed by the addition of either wild-type VP3 or mutant VP3, and VP7 sequentially with a 1.5 h incubation after each addition. The samples were then subjected to a 15% to 65% sucrose gradient, and the packaged RNA was quantified by qRT-PCR as previously described (23).

### vPAR-CL and click-seq

The method of vPAR-CL was previously described (13,24). BSR cells (for BTV infection) and MA104 cells (for RV infection) BSR cells (for BTV infection) and MA104 cells (for RV infection) were maintained in T175 flasks until 70–90% confluency. Cells were infected with BTV or RV at MOI = 1. 100 μM of 4-thiouridine (4SU, Sigma-Aldrich) was supplied twice to cells at 0 h p.i and 16 h p.i to reach a final concentration of 200 μM. For BTV, viruses were harvested at 48 hr p.i. and for RV, at 24 h p.i. BTV cores and RV DLPs were purified with methods described previously.

Cross-linking was conducted with a handheld UV device (3UV-38, UVP) with 0.15 J/cm^2^ of 365 nm at 4°C in dark room. Following cross-linking, capsids were digested with proteinase K and RNAs were extracted with previously described methods (13). The same procedures have been conducted on non-cross-linked 4SU containing viruses, which served as controls.

For testing BTV VP6 independently, we used purified rVP6 expressed by baculovirus expression system as described previously (18) and incubated with 4SU-labelled genomic RNAs extracted by standard phenol-chloroform extraction method from the labelled BTV cores. The rVP6 (200 μg/ml) was mixed with 200 μg/ml of 4SU-labelled purified RNAs in PBS and incubated at 28°C for 30 min before snap freeze.

ClickSeq libraries of cross-linked and control RNAs were constructed as previously described (13,25–27). Briefly, 250ng of RNA template was used in reverse transcription reactions using SuperScript III reverse transcriptase (Invitrogen) and standard conditions, with the exception of the supplementation of 3’-azido-nucleotides at a 1:35 AzNTPs:dNTPs ratio. Azido-terminated cDNAs were ‘click-ligated’ to Illumina i5 sequencing adaptors containing an additional 12N random nucleotides to act as a Unique Molecular Identifier (UMI). The click-linked cDNA was PCR amplified with barcoded Illumina p7 adaptors to generate final Illumina libraries. These were size-selected by gel electrophoresis on a 2% agarose gel and DNA 300–600 bp in length were extracted using Zymo gel extraction kits. Equimolar amounts of each indexed library were pooled and run on a NextSeq550 platform (Illumina) to yield 1 × 150 single-end reads and seven nucleotides for the index.

Three threshold filters were used to determine the significant and consistent signals for each experiment. In particular, each ‘U’ position has to meet the following criteria to be considered significant and consistent: (i) has a sequencing depth greater than 1000× in both control and crosslink samples; (ii) average vPAR-CL signal of the nucleotide must be greater than the mean signal (of the genome) + 1σ (representing ∼top 16% of signals); and (iii) has an increased U–C transition rate in at least 3/4 trials for BTV core, 2/3 for rVP6 and RV.

### Bioinformatics and data analysis

The Illumina sequencing data of vPAR-CL was subjected to the following bioinformatic pipelines, similar to previously described (13). Briefly, raw FASTQ data were prepared by trimming of Illumina adapter sequences, removing low quality reads and extracting UMIs with fastp (28): -a AGATCGGAAGAGC -U –umi_loc read1 –umi_len 14 –umi_prefix umi -l 30. Processed reads were subsequently mapped to the viral genomes using Bowtie2 (29). After mapping, PCR bias was de-duplicated with umi-tools : dedup –method = unique (30). Pile-up data was compiled using samtools mpileup (31) and subsequently analysed using ‘vPARCL-call’ (https://github.com/andrewrouth/vPARCL_call) to extract the nucleotide frequency at each genomic coordinate and to calculate the mismatch rate at each position, including the U-to-C transition rate, as expected in vPAR-CL data. The vPAR-CL signals are defined as the fold change of U-to-C transition rate between the cross-linked sample and the non-crosslinked control, as previously described (13). The prominent vPAR-CL sites were selected as both significant (with the average vPAR-CL signal greater than 1 standard deviation of mean) and consistent (has a sequencing depth greater than 1000 and an increased U-C transition rate compared to the control in at least 3 out of 4 replicates for BTV or 2 out of 3 for rVP6, RV).

### STREME algorithms analysis

RNA motif discovery was conducted by ‘STREME’ (32) which is provided by MEME SUITE v5.4.1 (33). The prominent vPAR-CL uridine sites along with 40 nts of flanking sequences (for a total of 41 nts.) were used as seeds for STREME assay. As a control, a list of at least 400 randomly selected sites of the correspondent genome and their flanking sequences (41 nts. In length) was used. For STREME assay, both seeds and controls were recognized as RNA (without considering complementary sequences), with motif length limited between 6 and 15 nts. and both p-value and E-value (accurate estimate rate) thresholds were set to 0.05.

## RESULTS

### Identification of the RNA interacting regions of the inner capsid layer of BTV core

**Figure 1.**
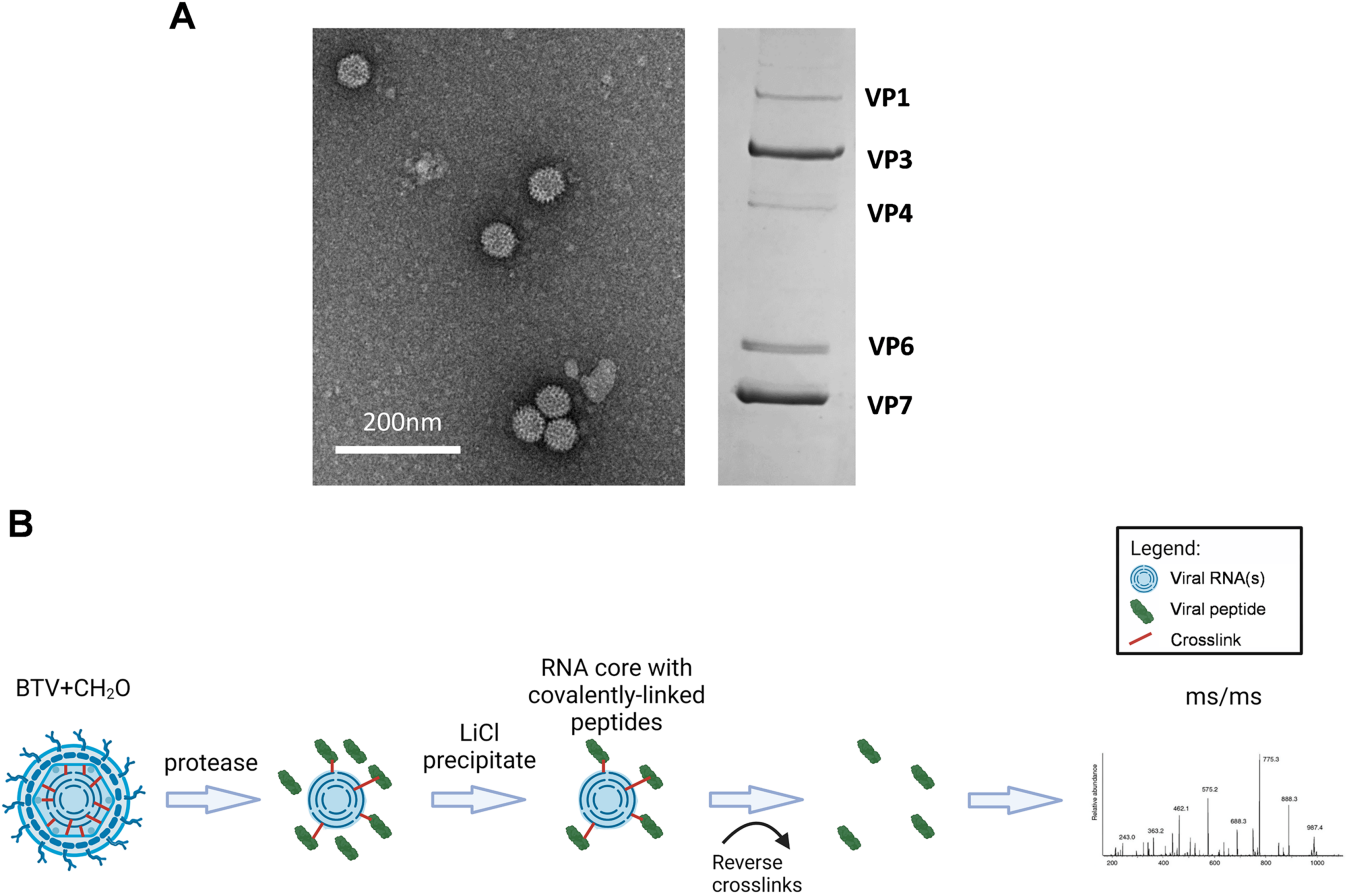
Purified BTV cores and RCAP workflow for the analysis of BTV cores. (**A**) Purified BTV cores are visualised via electron microscope and SDS-PAGE. BTV proteins are indicated. (**B**) The schematic for RCAP workflow. Each stage is indicated.

For some RNA viruses, genomic RNA and capsid protein interaction is critical for viral genome packaging and this interaction between viral capsid proteins and genomic RNAs occurs through preferred RNA binding sites (1–3,34). For BTV, it has been shown that VP6 and its interaction with the VP3 inner core layer is essential for packaging viral RNAs. However, it is not clear if VP3 directly interacts with viral RNAs and if so, such interaction influences virus replication. To address these questions, we used a proteomics-based RNA-cross-linking and peptide fingerprinting (RCAP) method, which had been highly successful at identifying RNA-VP6 binding regions in our previous study (9). Purified BTV cores composed of two structural proteins, VP7 and VP3, which encapsidate the replication complex (VP1, VP4 and VP6) (Figure 1A), together with 10 genomic dsRNA segments (S1-S10), were first cross-linked by formaldehyde before each viral protein–RNA complex was purified and subjected to trypsin digestion (Figure 1B). The resulting RNA-peptide complexes were then isolated from non-bound peptides and the peptide sequence analysed by collision-induced dissociation mass spectrometry. The majority of the peptides were from VP6, consistent with its role in BTV RNA packaging as reported previously (8,9,12). However, a number of peptides in the major inner capsid protein VP3, as well as other minor proteins, VP1 and VP4 were also identified. Importantly, these peptides were not identified in control reactions that were not subjected to formaldehyde crosslinking (Supplementary Table S2).

**Figure 2.**
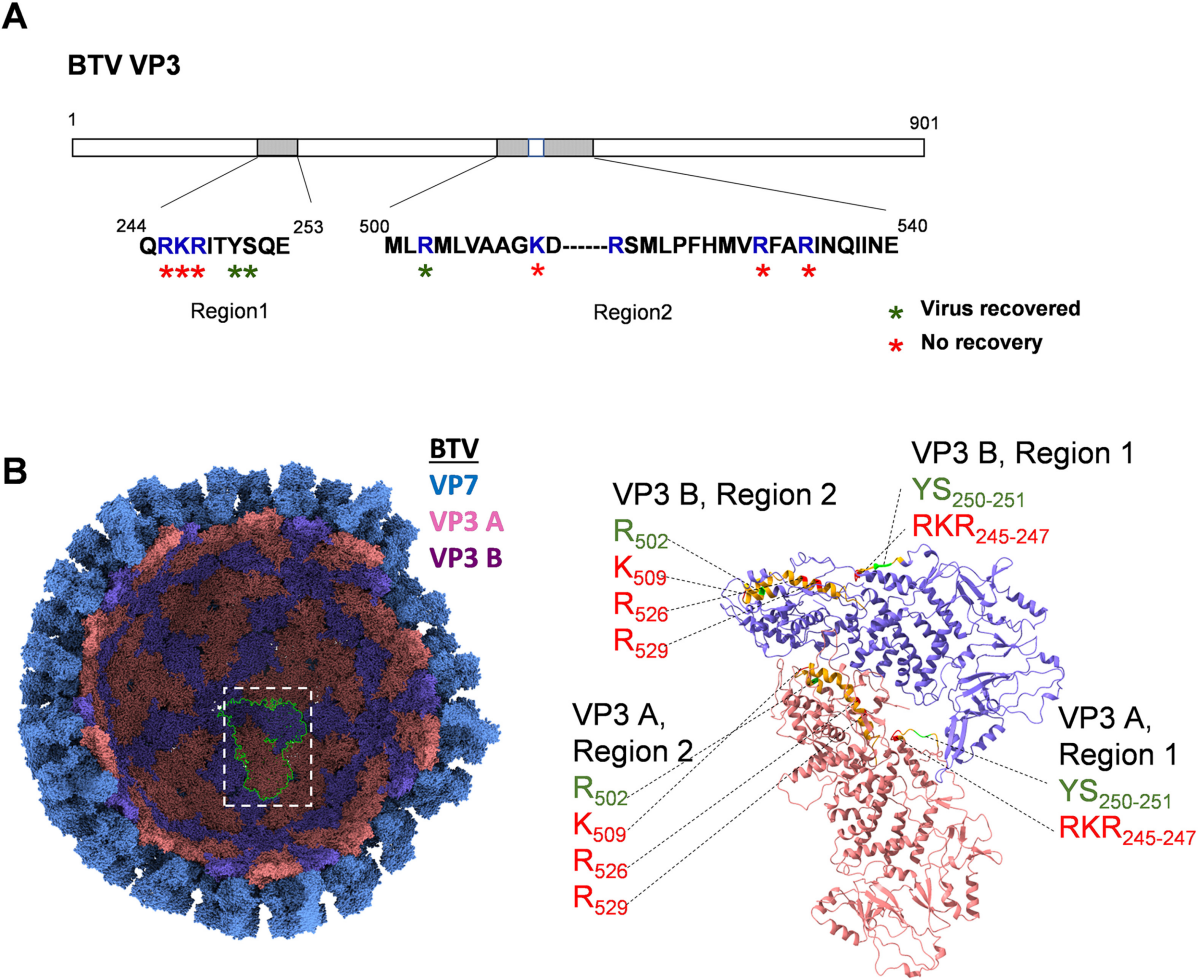
Visualisation of RNA-interacting regions on VP3 and results of virus recovery from key mutations. (**A**) RCAP identified RNA-interacting regions and amino acids are indicated. Blue letters indicate positively charged residues. The results of virus recovery after mutating the indicated residue are indicated. (**B**) A partial assembly of VP7 and VP3 for BTV solved by X-ray crystallography (2btv) are illustrated (left) with VP7 coloured blue, and Chain ‘A’ and ‘B’ from the asymmetrical dimer of VP3 coloured pink and purple respectively. In the icosahedral shell of the BTV core, 60 VP3 dimers form a convex disc shape with a series of shallow grooves (35). Ribbon models for VP3 A and B are illustrated (right) to indicate RCAP identified interacting regions (orange) in each subunit. Mutated amino acid residues that yield viable virus particles are coloured green, while mutated amino acid residues that failed to recover virus are coloured red. Amino acid identities and coordinates are indicated.

Peptides from two major regions of VP3 were identified being associated with the BTV genomic RNAs, one at residues 244 to 253 (VP3.Region1), and another at residues 500 to 540 (VP3.Region2) (Supplementary Figure S1A, Figure 2A). In the atomic structure of BTV core (35,36), monomers of VP3 forms a flat, elongated wedge shape structure with three distinct domains, a ‘carapace’ domain (aa 7–297, aa 588–698 and aa 855–901) which forms a rigid plate representing the majority of the inner surface of the VP3 capsid, an ‘apical’ domain (aa 298–587), situated closest to the fivefold axes in the intact particle and a ‘dimerization’ domain (aa 699–854), which is situated furthest away from the fivefold axes. The VP3.Region1 and VP3.Region2 are located where the carapace domain contacts the apical domain (Supplementary Figure S1B, Figure 2B).

### Mutational analysis of VP3 affects capsid formation and RNA packaging.

**Figure 3.**
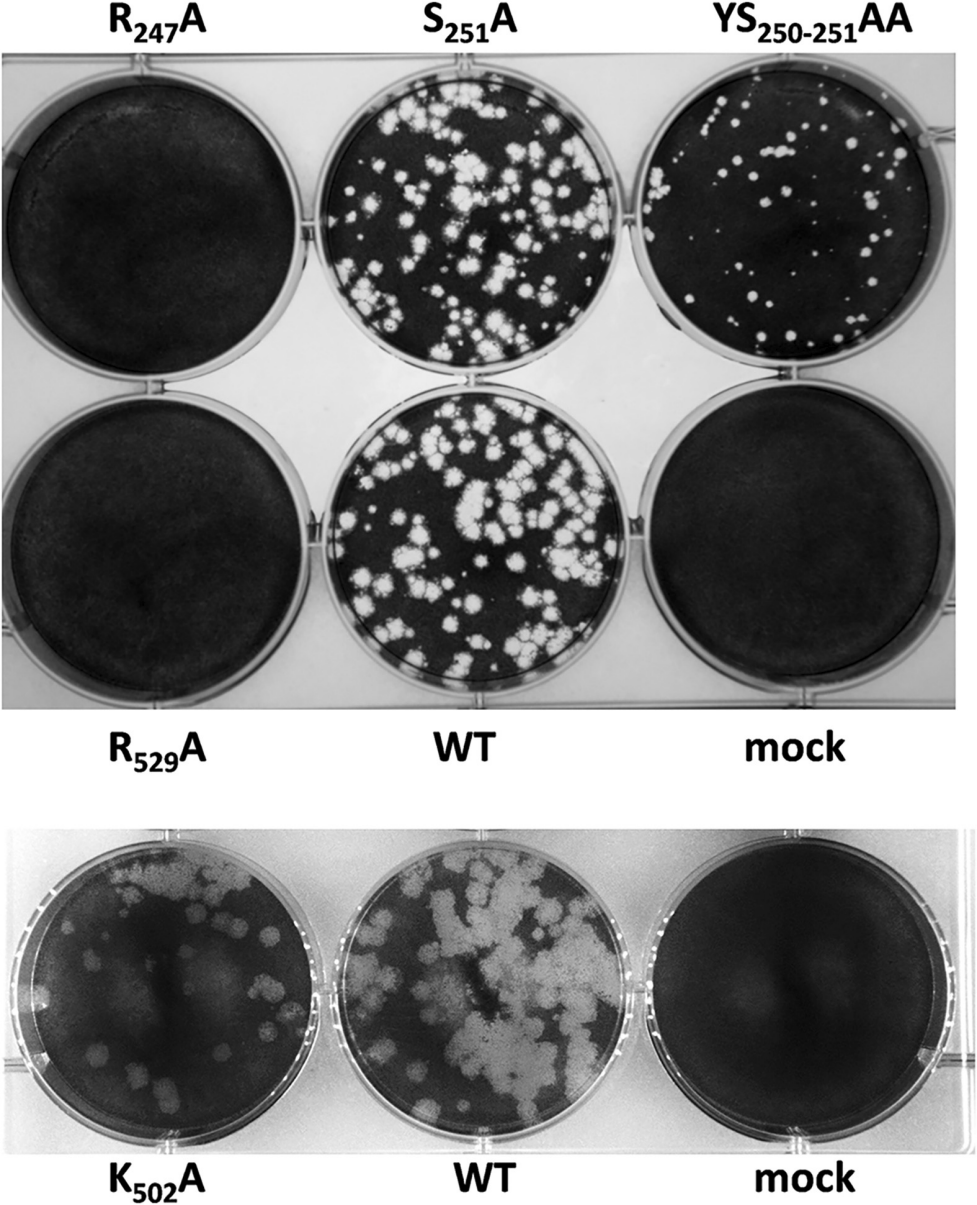
Plagues of VP3 mutant viruses. Different VP3 mutants, Wildtype (WT) and mock infection are indicated.

VP3.Region 1 contain an arginine, lysine and arginine at position 245–247 that are highly conserved among all Orbiviruses (Supplementary Figure S2A). VP3.Region 2 has five positive charge residues that are dispersed. This region is also genetically conserved (Supplementary Figure S2A), indicating it has potential functional importance in virus replication. Since positively charged residues are most likely to interact with negatively charged phosphate backbone of genomic RNA, we mutated the nucleotides in S3 that encodes RKR245-247 to encode alanines (RKR245-247AAA). To determine if these substitutions had any impact in virus replication, the mutated genomic RNA was then subjected to virus recovery using the BTV reverse genetics (RG) system (21). However, virus recovery was unsuccessful (Figure 2A). To determine whether changing only one of these three residues would also have an impact on virus replication, a single alanine substitution mutant RNA at residue 247 (R247A), was created, which also failed to recover viable virus (Figures 2A, 3). To support this data, a second set of mutations was generated targeting the adjacent uncharged residues, a single mutation, S251A and a double mutation YS250-251AA. When these mutant RNA genomes were subjected for virus recovery, both mutant genomic RNAs successfully recovered viable viruses. While the single substitution, S251A, had no observable effects on phenotype as the virus grew at a similar rate to wild type, the double mutant, YS250-251AA, generated smaller plaques indicating a level of attenuation (Figure 3). Each mutant genome was confirmed by sequencing. These results suggest that residues RKR245-247 in VP3.Region 1 are important for virus replication.

**Figure 4.**
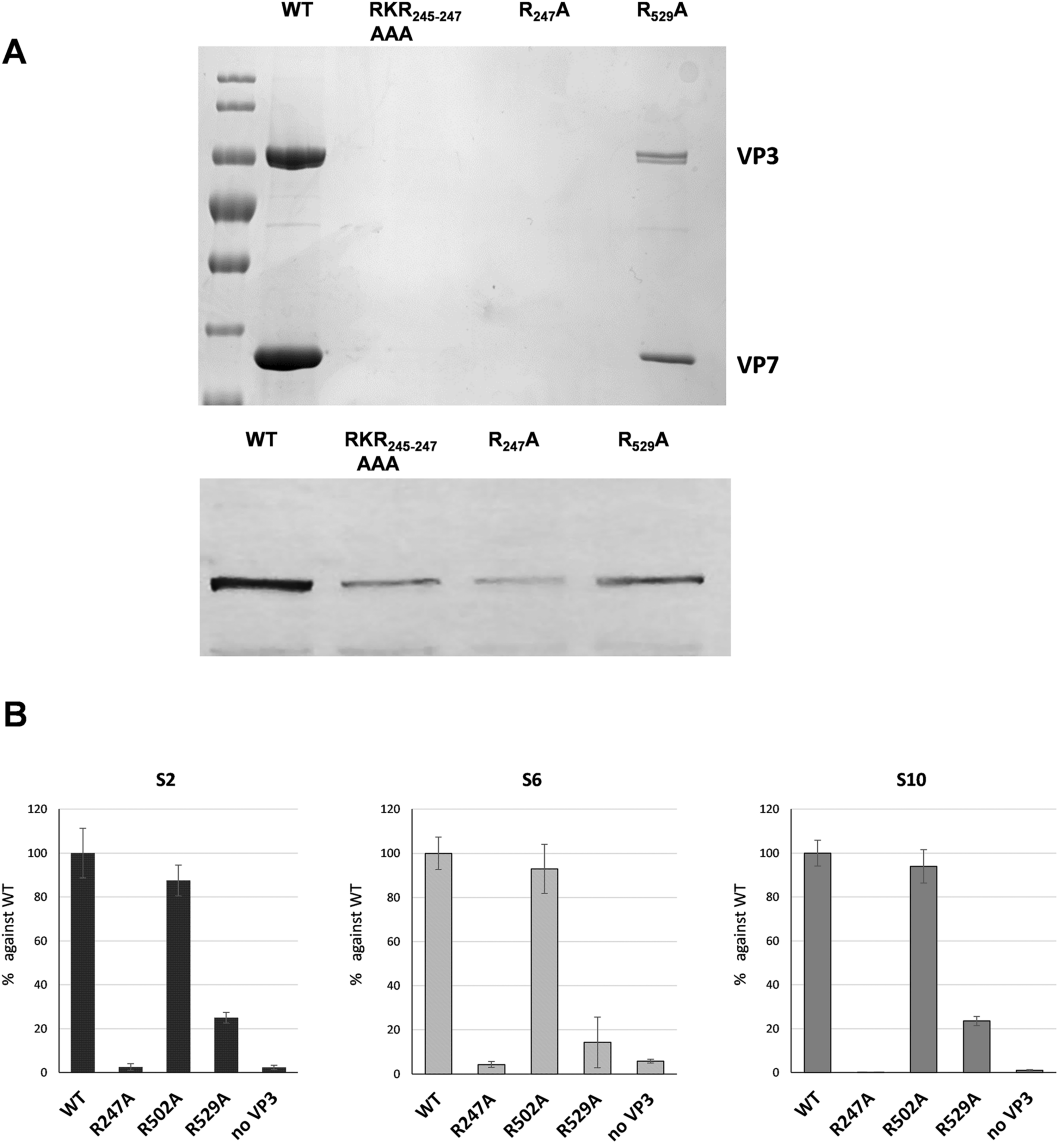
Capsid formation and RNA packaging from different mutations on VP3-RNA interaction sites. (**A**) Upper panel: Different CLP samples analysed by SDS-PAGE. The VP3 and VP7 bands are indicated. Lower panel: VP3 specific immunoblotting shows the expression of mutant proteins. (**B**) CFA assay was performed using wild-type VP3 (WT), R274A mutant VP3, R529A mutant VP3, or without VP3 (no VP3). The BTV RNA (segments S2, S6 and S10) packaged and protected after RNase treatment was quantified and the average quantities of three repeated experiments are shown.

For VP3.Region2, four highly conserved positively charged residues (R502, K509, R526 and R529) were selected for mutagenesis. Of the 4 mutants, only mutant R502A yielded virus, suggesting that residues K509, R526 and R529 play an important role in virus replication. To clarify whether the failure of virus recovery is due to interference with RNA-capsid interactions, or structural changes of the icosahedral capsid, we verified capsid assembly using the recombinant core-like particles (CLPs) system (19,37). In brief, a recombinant baculovirus was generated to express mutant VP3, which was then used to co-infect insect cells together with a recombinant baculovirus expressing wild-type VP7 to generate stable CLPs (37). We selected three of the mutants that not generate viable virus particles (RKR245-247AAA, R247A and R529A) for CLP assembly. This assay demonstrated that the R247A and RKR245-247AAA VP3 mutants were expressed as recombinant proteins, albeit at reduced amounts, but did not form CLPs (Figure 4A). Changing RKR245-247 to alanine thus affected the structural integrity of VP3 and/or its interaction with each other or with VP7. However, the results were different with the R529A VP3 mutant. Although it failed virus recovery, could still form CLPs indicating that the lethal effect on virus replication was likely due to failure of its interaction with RNA.

To investigate this further, we performed an *in vitro* cell-free assembly (CFA) assay which allows the generation of infectious core particles with a full complement of 10 RNA segments (11,23). In brief, 10 BTV genomic RNA segments were pre-incubated with VP1, VP4 and VP6 followed by incubation with either wtVP3, R247A mutant VP3 (incapable of capsid assembly), R502A mutant VP3 (capable of virus replication), R529A mutant VP3 (capable of capsid assembly) or no VP3. The RNA-protein complexes were then incubated with VP7 to form stable double-layered capsids. Under RNase treatment, RNA can only be detected following successful packaging and protection within the capsids. Since the 10 segments are packaged sequentially, from smaller to larger segments (23), after RNase treatment, we analysed representative RNA segments of three different sizes, S2 (a larger segment), S6 (a medium segment), and S10 (a smaller segment). In this assay, the wild-type capsids and the capsids produced with R502A, which did not impact virion formation, had high level of protected RNAs. R247A VP3 mutant, which disrupted VP3 capsid formation, perturbed RNA packaging to the same level observed when VP3 was not present (Figure 4B). R529A, however, caused significant reduction of RNA packaging by up to 75% (Figure 4B). These results suggest that the mutation of R529 at the apical domain, inhibited RNA packaging despite its ability to form capsid structure.

### Investigation of genomic RNA interacting sites of the encapsidated BTV polymerase complex proteins, VP1 and VP4

In addition to VP3 and VP6, there are two additional proteins inside the core, VP1 (1302 residues), which functions as an RNA dependent RNA polymerase (RdRp) and VP4 (644 residues), which modifies the 5’ termini of mRNAs prior to their release from the viral core (38,39). High-resolution structures of both proteins are available (40,41). Due to the flexible nature of the position of these two proteins in the viral core, it was difficult to obtain precise RNA interaction data. Nevertheless, it was clear that VP1 and VP4 also interact with genomic RNA in the core as suggested by RCAP.

**Figure 5.**
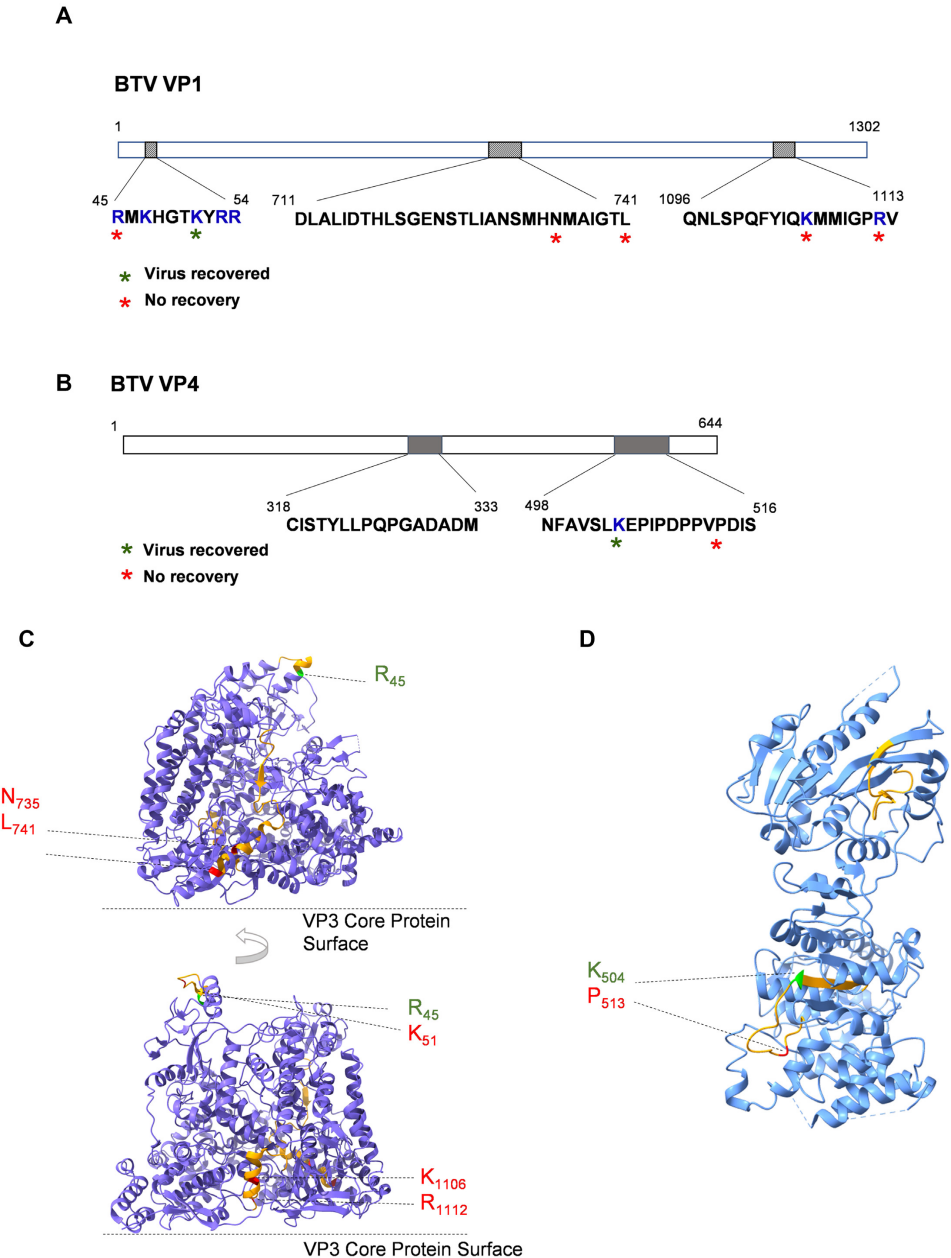
RNA-interacting regions in BTV VP1 and VP4. RCAP identified RNA-interacting regions and amino acids of BTV VP1 (**A**) and VP4 (**B**) are indicated. Blue letters indicate positively charged residues. The virus recovery results after the indicated residues mutated are indicated. (**C**) Ribbon model for BTV VP1 solved by cryo-EM (6pns) is illustrated (two different side views) to indicate RCAP-identified RNA-interacting regions (orange). Mutated amino acid residues that yield viable virus particles are coloured green, while mutated amino acid residues that failed to recover virus are coloured red. Amino acid identities and coordinates are indicated. (**D**) Ribbon model for BTV VP4 solved by X-ray crystallography (2jhc) is illustrated to indicate RCAP-identified RNA-interacting regions (orange). Mutated amino acid residues that yield viable virus particles are coloured green, while mutated amino acid residues that failed to recover virus are coloured red. Amino acid identities and coordinates are indicated.

VP1 is composed of three domains, the N-terminal (aa 1–387), polymerase (aa 388–935), and C-terminal bracelet domain (aa 936–1302) (36,41). RCAP analysis of VP1 revealed three distinct RNA- binding regions which are distributed across all three domains (Figure 5A). One region (aa 45–54) is located in a unique helix bundle within the N-terminal domain, and is close to the terminal RNA (34) (Figure 5C). This region is rich in positively charged residues, with five of the ten residues being either arginine (R) or lysine (K) amino acids. The second region, aa 711–741, is located within the central polymerase domain, at the base of the fingernail subdomain, stretching into the palm subdomain. This region is located 20 residues upstream of the signature RdRp GDD motif (aa 763–765 for BTV) (41) (Supplementary Figure S2B). The third interacting region, aa 1096–1113 is situated at the C-terminal bracelet domain. To test the importance of these three regions for virus replication, we selected six residues in these three regions (R45, K51, N735, L741, K1106 and R1112) for virus recovery via the RG system. Each mutant genomic RNA was assessed for virus recovery and the results showed that out of six sites targeted, only K51A was able to recover virus (Figure 5A; Supplementary Figure S3).

The capping enzyme VP4 contains four domains, the N-terminal KL domain (aa 1–108), the (guanine-N(7)-)-methyltransferase (N7MTase) domain (aa 109–174 and aa 378–509), the (nucleoside-2’-*O*-)-methyltransferase (2’ OMTase) domain (aa 175–377), and the C-terminal guanylyltransferase (GTase) domain (aa 510–644). The atomic structure of VP4 has been solved using BTV VP4 monomers expressed in insect cells (40). However, the *in-situ* conformation of the oligomeric form or the exact location of VP4 within viral core has yet to be elucidated. Understanding the interaction between VP4 and BTV RNA, especially with the genomic dsRNAs within the viral capsid, might not only help to clarify its location within the viral cores but may contribute to our understanding of how VP4 function is initiated during RNA transcription *in situ*. Two RNA interacting regions were identified via RCAP analysis (Figure 5B), aa 318–333 is located in the 2’ OMTase domain and aa 498–516 at the end of the N7MTase domain and the beginning of the GTase domain, including part of the very highly conserved motif PDxSLCRFxGL (aa 513–523) in GTase domain (38) (Figure 5D; Supplementary Figure S2C). To test whether the conserved motif residues played an important role, P513 was substituted for a glycine. The data obtained by virus recovery demonstrated that the P513G mutant could not be recovered (Figure 5B; Supplementary Figure S3), indicating the critical role of this site.

### Identification of RNA sequences that are responsible for interaction with BTV proteins

**Figure 6.**
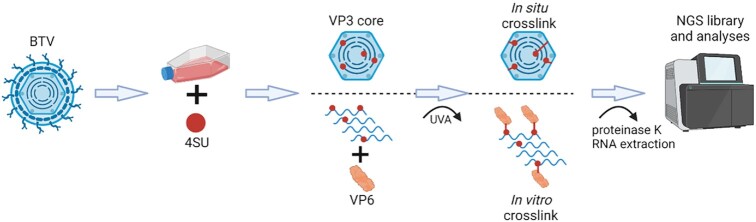
vPAR-CL workflow for the analysis of genomic RNA with BTV core or BTV rVP6 protein. Each stage is indicated.

BTV RNAs are specifically recognised and packaged into the viral core (11,42). However, it is not clear which genomic segments interact to those capsid proteins or the specific nucleotide sequences involved. It is still also unclear whether the *Reoviridae* viral genomic dsRNA segments occupy fixed positions within the core. If this is the case, then certain sequences of the genomic RNA would specifically interact with capsid proteins to stabilize the orderly packaging of genomic segments within capsid. To clarify this, and to identify the RNA sequences that specifically interact with viral proteins, we used viral photoactivatable ribonucleoside cross-linking (vPAR-CL) method (13,24). In brief (Figure 6), BSR cells infected with BTV were supplemented with 4-thiouridine (4SU), which is metabolised intracellularly into 4-thiouridine triphosphate and subsequently incorporated into the newly synthesised genomic RNA segments. The 4SU-labelled RNA segments within the capsid and any capsid protein close to them were then UV cross-linked as described in the methods. The BTV core preparation was highly purified making the only reactants the BTV capsid proteins and genomic RNAs although trace amounts of host material on the outside of the capsids cannot be ruled out. However, the predominant cross-linking is expected to occur between viral proteins and viral genome inside the capsid.

**Figure 7.**
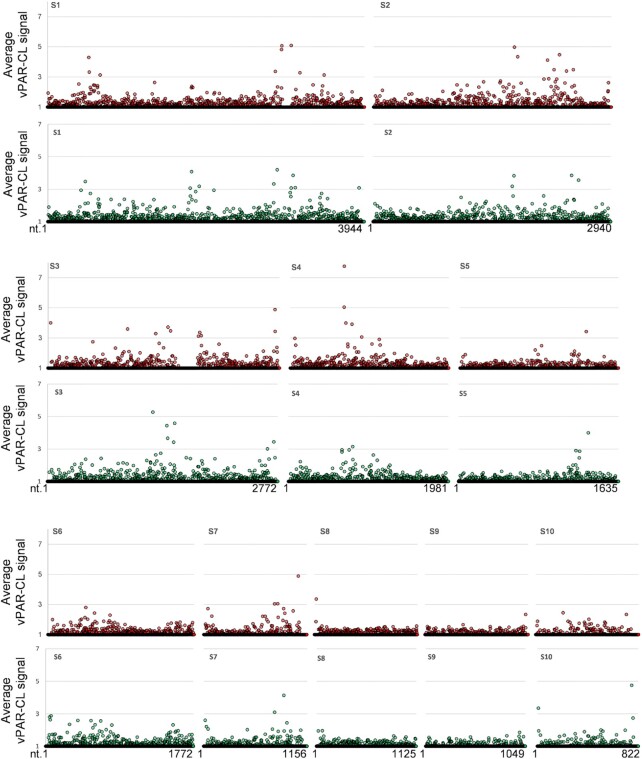
The average signal results from vPAR-CL for BTV RNAs. The average signals of the 10 segments of 4SU enriched BTV genomic RNA interacting with viral proteins in BTV cores (red) or rVP6 *in vitro* (green) are individually shown.

**Figure 8.**
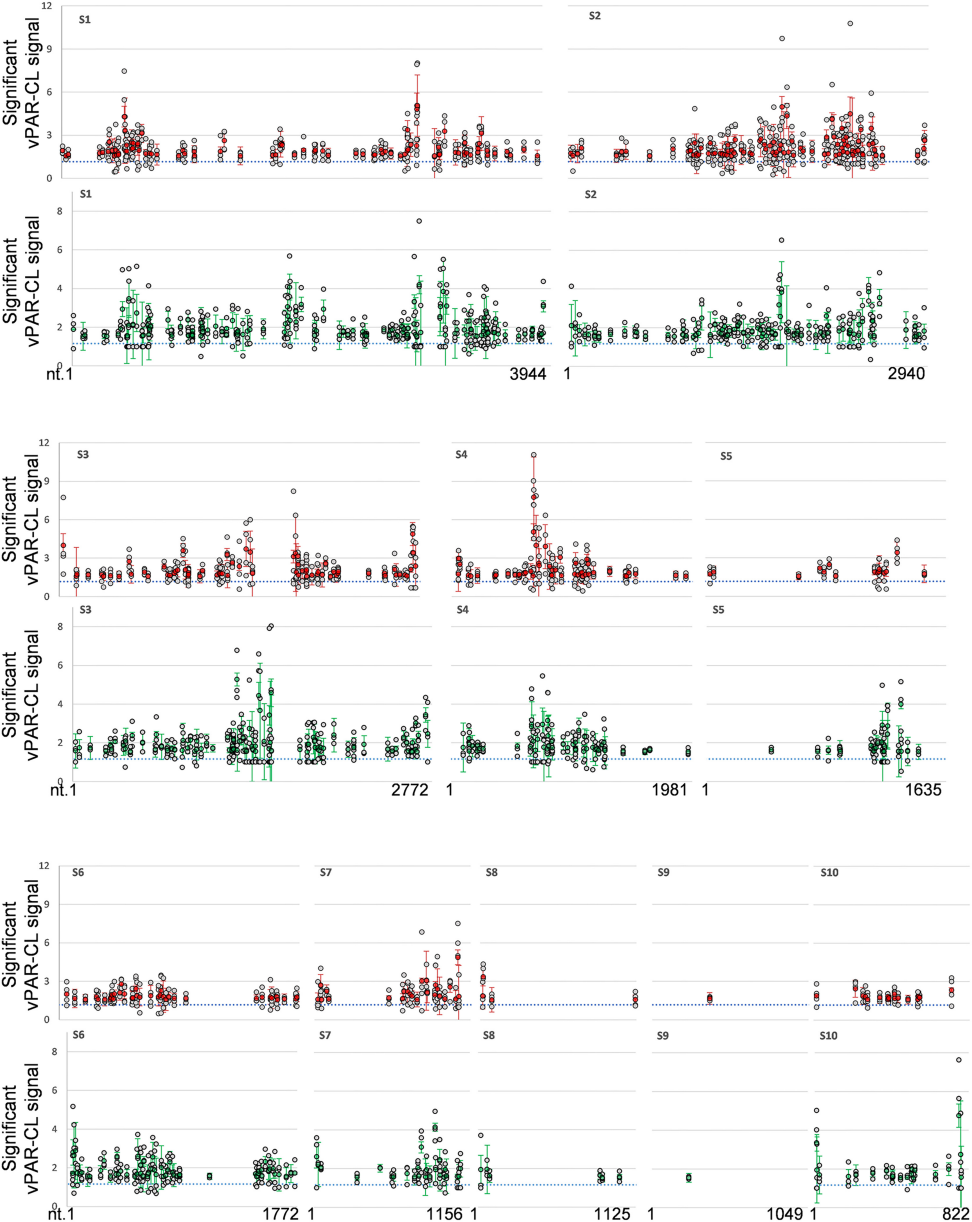
The Prominent protein interacting sites identified by vPAR-CL for BTV RNAs. Red = average signal of BTV core, green = average signal of rVP6, grey dots = individual repeat, error bar = stdev, blue dotted line = average signal of all genomic U positions.

**Figure 9.**
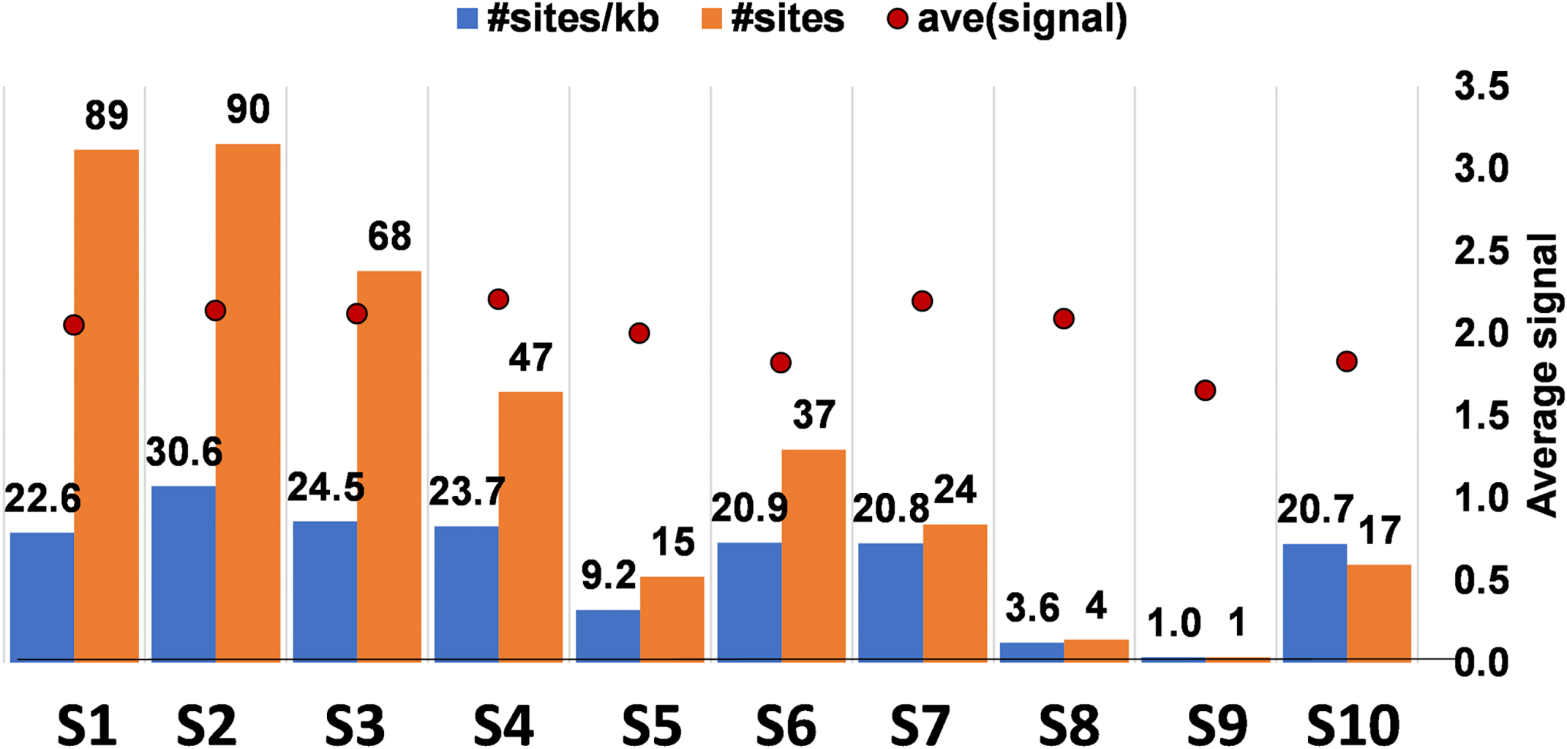
The distribution of protein interacting sites in genomic RNAs within BTV cores. The numbers of protein interacting sites (#sites) and ratios of sites per kb (#sites/kb) on each segment are shown.

The viral protein-interacting RNA sites are indicated by vPAR-CL signals, which are defined here as the fold change of U-C transition between the cross-linked (4SU+/UV+) experiment and non-cross-linked (4SU+/UV-) control. From the average vPAR-CL signals of four replicates, RNA-protein interacting sites were readily detectable across the entire BTV dsRNA genome, with extensive RNA-protein interactions sites located within the inner core layer (Figure 7). However, the RNA-protein interacting pattern was not uniformly distributed since certain genomic regions showed dense clusters of signals while others did not. Clear discrepancies of signal distribution could also be observed among different genomic segments of BTV. This became more apparent when the data was further filtered to highlight the sites that are both significant (average vPAR-CL signal greater than 1 standard deviation of mean) and consistent (has a sequencing depth greater than 1000 and an increased U-C transition rate compared to the control in at least 3 out of 4 replicates) (Figure 8). We observed a clear distinction between genomic segments (Figure 9) and that the distribution of significant and consistent vPAR-CL sites had little correlation with the length of the respective genome segments, with 7 out of the 10 segments containing ∼20–30 significant vPAR-CL sites per kb genome, despite their differences in length.

Among these, the second largest segment (S2, FJ969720.1, 2940 nts.) contained the greatest number of significant sites (90 sites, 30.6 sites/kb), which is noticeably more than contained in the largest segment (S1, FJ969719.1, 3944 nts.). The smallest segment (S10, FJ969728.1, 822 nts.) contained 17 significant sites (20.7 sites/kb), in stark contrast to other smaller segments such as S8 (FJ969726.1, 1125 nts., 4 sites) and S9 (FJ969727.1, 1049 nts., 1 site). This demonstrated that the encapsidated dsRNA segments of BTV exhibit a fixed topology which provides different levels of RNA-protein interaction inside the core. It is likely that certain segments of the genome (S8, S9) may be organized more centrally and occluded by other genomic segments, since both were observed to contain the least amount of RNA-protein interaction sites. In contrast, the larger segments (e.g. S1, S2) and the smallest S10 may be more accessible in order to support an increased number of organized viral protein–RNA contacts inside the core.

### Identification of the RNA sequences that interact with VP6 *in vitro*

BTV inner core contains several proteins (VP3, VP1, VP4, VP6) that could interact with encapsidated dsRNA genome. Among these, VP6 is known to have strong RNA binding affinity (16,40). To distinguish the VP6 binding sites from the other inner core proteins, the same vPAR-CL method was performed *in vitro*, with purified recombinant VP6 (rVP6) and 4SU labelled dsRNA extracted from BTV cores (Figure 6). The rVP6 and labelled genomic RNA were incubated *in vitro* to form RNA-protein complexes and were UV cross-linked as described in the methods (9).

The vPAR-CL signals of BTV dsRNA with rVP6 generally agreed with those observed for the core (Figure 7). The vPAR-CL signals of BTV dsRNA with rVP6 generally agreed with those observed for the core (Figure 7). Again, the protein–RNA interaction sites that are significant (average vPAR-CL signal greater than 1 standard deviation of mean) and consistent (increased U–C transition rate compared to the control in at least two out of three replicates for sites with sequencing depth >1000) interacted with rVP6 *in vitro* (Figure 8). The distribution of these rVP6 sites across segments was similar to that for BTV core, with the smaller segments S8 and S9 having fewer interacting sites, compared to the larger segments (Supplementary Figure S4).

**Figure 10.**
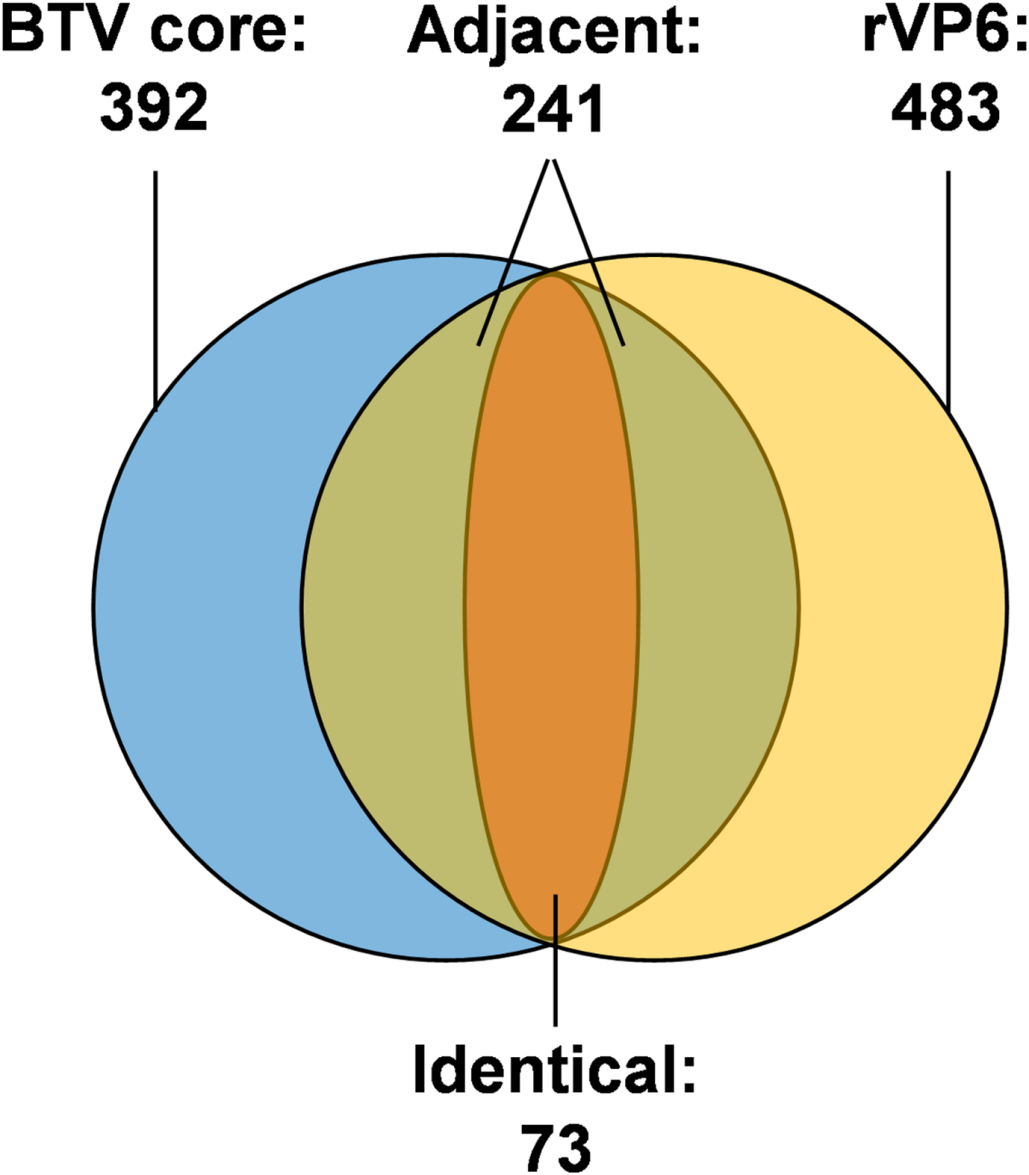
The distinct and overlapping protein interacting sites between BTV core and rVP6. Blue and yellow circles indicate the protein interacting sites identified from BTV cores and rVP6 within the whole BTV genome, respectively. The orange indicates the identical sites. Identical: co-discovery of the same sites in both BTV core and rVP6 datasets. The green and orange regions together indicate adjacent sites. Adjacent: 1 or more VP6 sites were found within 10nts from a BTV site.

A comparison of the significant and consistent sites between BTV core (392 sites) and rVP6 (483 sites), found that ∼19% of interaction sites in the core are identical to those found in rVP6 (73 sites), and ∼61% sites in the core are adjacent (within 10nts) to those found in rVP6 (241 sites, including identical ones) (Figure 10; Supplementary Figures S5, S6). The heterogenicity of crosslinked 4SU sites within the same viral protein–RNA complex has previously been demonstrated (13). It is conceivable that the majority of discovered BTV dsRNA-protein interactions (in capsid) represent the same RNA–protein complexes as those found in rVP6 in the *in vitro* binding system. Although VP6 may not the only protein partner that interacts with encapsidated genomic RNA segments, these data indicated that VP6 plays a significant role in regulating RNA packaging and anchoring each RNA segments within the capsid.

### Does capsid selectively interact with a motif in the RNA segments?

**Figure 11.**
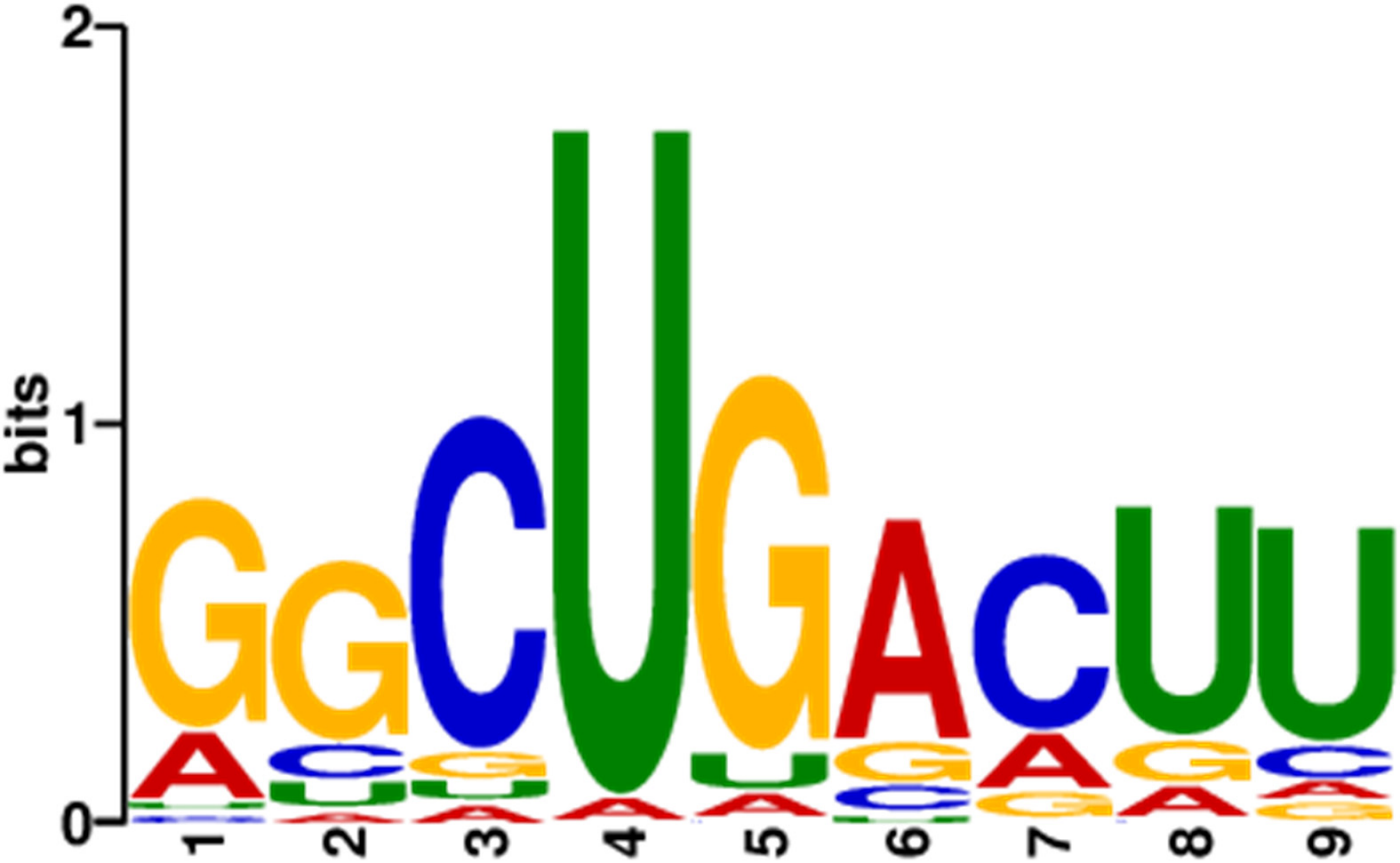
The STREME predicted 9 base protein interacting motif for BTV. STREME analysis of vPAR-CL sites in BTV core discovered a significant RNA motif (p-value: 7.5e-3, E-value: 3e-2) with GU rich sequences.

The specific interaction between capsid and RNA must be achieved through a certain level of selective binding. Since there are limited viral proteins within the viral capsid, we hypothesized that viral proteins recognise similar RNA sequences or secondary structures. We further investigated whether there was any sequence similarity among the many vPAR-CL sites that indicate extensive BTV dsRNA and viral protein interactions, across the different RNA segments. All the prominent vPAR-CL sites, along with flanking sequences (41nts. in length, centralized crosslinked U) were analysed with the ‘STREME’ computational pipeline (32) to identify potential motifs in the RNA sequence that are continuous, recurring and of fixed-length. We first compared the prominent vPAR-CL sites of the BTV core as seeds (392 sequences, average length 39.9 nts.) with a set of randomly selected control sequences from the BTV genome (400 sequences, average length 39.9 nts.). This analysis identified one sequence motif ‘GGCUGACUU’ (Figure 11) that was enriched in the seed sequences relative to the controls (*P*-value: 7.5e–3, *E*-value: 3e–2) and occurred at multiple locations across different genome segments (Table 1).

**Table 1.** STREME predicted motif in BTV genomic RNAs. The vPAR-CL identified sites which bind to BTV core and have more than 6 matches to the STREME motif are listed. The nucleotides in the site are underlined and matching nucleotides are marked in bold. The sites which have more than 7 matches were tested for virus recovery and the results listed. Attenuated indicates that the virus can be recovered but has a reduced growth rate. The sites which are adjacent to rVP6 interacting sites (<10 nt. distance) are listed

Segment	Start of motif	Sequence (**motif**)	Virus Recovery	Adjacent w/ rVP6 sites
**S1**	523	AGUUAU**GGCUGACUU**UGUAAACCGUUUCGA	No	Yes
**S1**	2771	CCUAUA**GGCUG**GAA**U**GGGUACGGCGCGCAU	-	Yes
**S1**	3541	GAAGAGCUA**GCUGA**A**U**AUAUGACUUCAGAA	-	No
**S2**	1305	AUUGUUCAUUGGGAAUAUA**GGCUGA**A**UC**AU	Attenuated	Yes
**S2**	1704	CCAAUCGAAUUGCAAA**GG**U**UGACUU**UGGCG	Attenuated	Yes
**S2**	1141	GAAGCUA**G**G**UGAC**G**U**UUAUUCGAUGAUGCG	-	No
**S3**	1828	AUGC**G**U**CUGA**G**UU**AUCCGUGAUGAAGGUAG	No	Yes
**S3**	1488	CAUAUGGCAUUG**GG**A**UGACUU**AUCAUUGCU	–	Yes
**S3**	2617	GACAGAUUCU**GGCUG**UA**UU**GAACAGAAGAU	No	Yes
**S3**	2700	GAUGGGCACCAA**GCUG**G**CUG**CGCCAACUGU	-	No
**S4**	601	UACGUUGGAUGC**G**G**UGACUU**ACGUACCUUG	No	No
**S6**	274	UGAUCGGGAGACA**G**U**CUG**G**CU**CCAAUGCUU	-	Yes
**S7**	796	UUAAAUCAAUACC**CUG**C**CUU**GACUGCUGAA	-	Yes
**S7**	988	CUGUUUUAAGAC**CUGA**G**UU**CGCGAUCCACG	-	Yes
**S7**	936	ACCAAACGAUCGAGAUAGUAUC**CUGACUC**U	-	Yes
**S10**	243	GAA**GGCUG**CA**UU**CGCAUCGUACGCAGAAGC	Yes	No
**S10**	537	UUGGG**GGCU**AC**CUU**CCUGAUGAUGGUCUGCG	No	Yes

Among these sites that potentially contain the specific motif, we tested the sites which had at least 7 matching nucleotides for virus recovery. The nucleotide sequences were carefully changed without altering amino acid coding sequences. Of these mutations, nt523-531 of S1, which was an exact match for the predicted 9 nucleotide motif sequence, was lethal to the virus. Similarly, S3 nt1828-1837, S3 nt2617-2626, S4 nt602-611 and S10 nt538-547, which all matched 7 nucleotides with the proposed motif, were also shown to be critical for virus replication. After altering the nucleotide sequences of S2 nt1305-1314 and S2 nt1704-1713, virus could be recovered, however the mutant virus had a reduced growth rate (Supplementary Figure S7). Among the tested sites, only S10 nt243-252 had no impact upon virus recovery. The S3 nt1488-1497 has eight nucleotides matching the motif sequence, however it was not possible to make the required nucleotide changes without altering the amino acid sequence therefore this site was not tested for virus recovery.

When a similar motif-finding approach was applied to the vPAR-CL sites bound by rVP6, no sequence motif was identified which passed the set threshold of both p-value and E-value in the dataset. It is noteworthy, that although there is no motif identified from the sites bound to rVP6, the sites matched to the predicted motif are mostly (12 out of 17) identical or adjacent with the sites bound to rVP6 (Table 1, Figure 10), indicating that VP6, may only play a supporting role in RNA motif recognition within virus capsid.

### Protein-RNA interaction in rotavirus capsid and the RNA sequences of genomic segments that interact with the rotavirus capsid proteins

RV and BTV both belong to *Reoviridae* family and share many similarities. The inner core/capsid structure of these two viruses are very similar and it was proposed that they share similar RNA packaging mechanism (36,43). Similar to BTV, RV inner capsid contains a major capsid protein (VP2), a RdRp (VP1) and a capping enzyme (VP3). The major difference between these two viruses is that BTV and other orbiviruses have an additional RNA packaging protein, VP6, which interacts with both RNA and the VP3 capsid protein (9,12) while RV lacks the equivalence of this specific protein. However, the structural data demonstrated that RV VP2 is associated with genomic RNA. Therefore, for comparison with BTV, we investigated the interactions between the RV capsid proteins and genomic RNA using RCAP.

**Figure 12.**
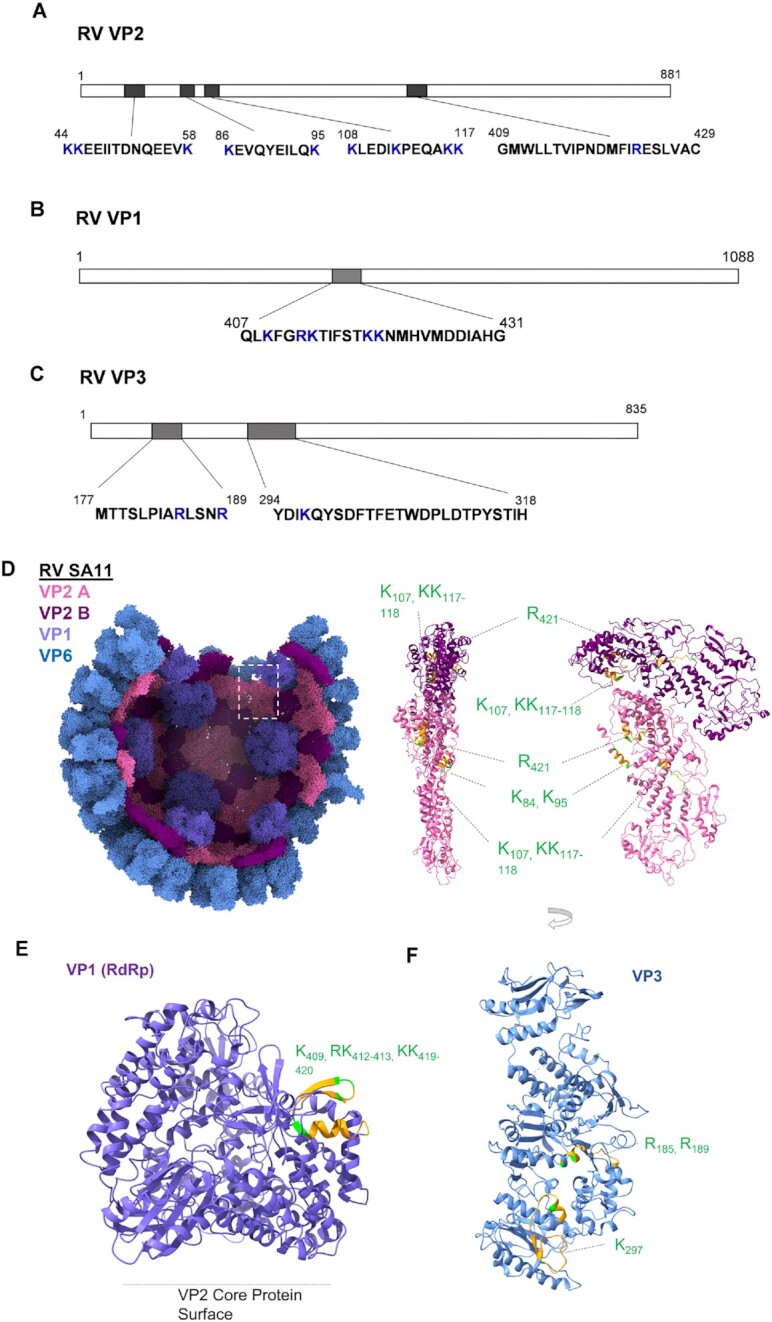
RNA-interacting regions in RV VP2, VP1 and VP3. The RCAP identified RNA interacting-regions and residues in RV SA11 capsid protein VP2 (**A**), VP1 (**B**) and VP3 (**C**) are indicated. Positively charged residues within the regions are marked as blue. (**D**) A partial assembly of VP2, VP1 and VP6 RV solved by cryo-EM (6ory) are illustrated (left) with chain ‘A’ and ‘B’ from the asymmetrical dimer of VP2 coloured light pink and hot pink respectively, VP1 coloured purple, and VP6 coloured blue. Ribbon models for VP2 A and B are illustrated (right) (two side views) to indicated RCAP identified RNA-interacting regions (orange) in each subunit. (**E**) Ribbon model for RV VP1 solved by cryo-EM (6ory) is illustrated to indicate RCAP-identified RNA-interacting regions (orange). Positively charged residues within the regions are coloured green. (**F**) Ribbon model for RV VP3 solved by cryo-EM (6o6b) is illustrated to indicate RCAP-identified RNA-interacting regions (orange). Positively charged residues within the regions are coloured green. Amino acid identities and coordinates are indicated.

Unlike the BTV core, where the majority of the RNA interaction was with VP6, the majority of the RNA interaction in RV was with VP2, the major capsid protein (Supplementary Table S3). VP2 has four distinct domains: N-terminal domain (NTD), apical domain, carapace domain and dimer-forming domain. RCAP analysis of the RV double layer particles (DLPs) revealed that VP2 contacted the encapsidated RNAs at four regions: aa 44–58, aa 86–95, aa 108–117, and aa 409–429 (Figure 12A, D). The first two are located within the NTD which was previously reported to interact with the RdRp (43). The third is located in the carapace domain next to the NTD whilst the fourth is located in the apical domain. Notably, the BTV capsid protein VP3 does not contain RNA-interacting regions in the N-terminal region. This indicates that the interaction between RV VP2 N-terminus, RdRp, and genomic RNAs could be a unique mechanism for RV. The fourth region in RV VP2 shares similar structural location to BTV VP3.Region2.

The RCAP analysis also revealed that unlike BTV VP1, which harbours three RNA interacting regions, only one region in RV VP1 (aa 407–431) interacts with genomic RNA. This region is located in the finger's subdomain of the polymerase domain (Figure 12B, E), which is more upstream compared to the BTV VP1 (44). In addition, two genomic RNA interacting regions (aa 177–189, and aa 177–189), were identified in the VP3 capping enzyme of RV (Figure 12C, F) at the equivalent positions to that of the BTV capping enzyme VP4.

**Figure 13.**
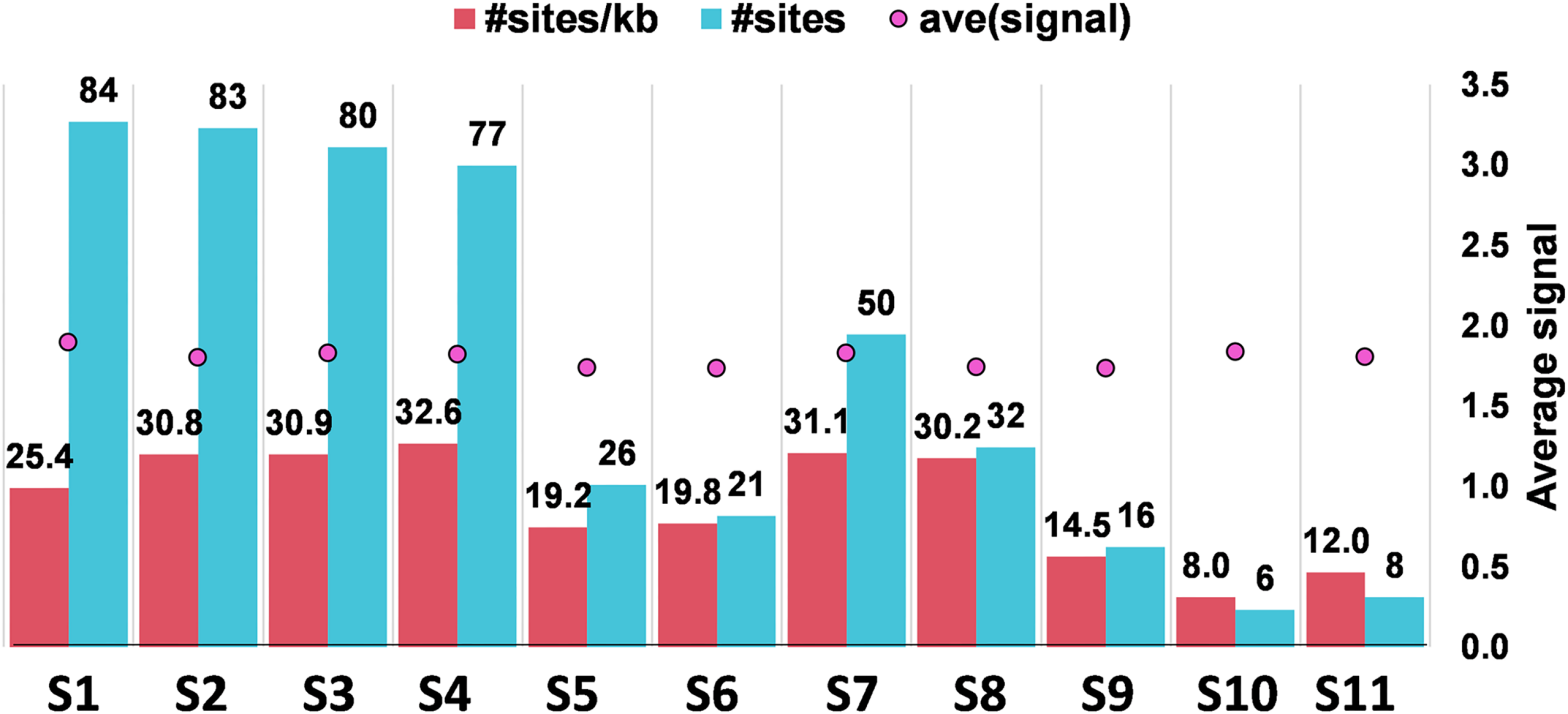
The distribution of protein interacting sites in genomic RNAs within RV DLPs. The numbers of protein interacting sites (#sites) and ratios of sites per kb (#sites/kb) on each segment are shown.

Subsequently, we carried out vPAR-CL experiments in RV (SA11 strain) using highly purified RV DLPs, which revealed that across the 11 genome segments of RV, an uneven distribution of vPAR-CL sites was observed (Supplementary Figure S8). Clusters of sites could be noted in all 11 genomic segments of RV. The vPAR-CL sites appeared to favour the termini of each genome segments, but prominent signals could also be found in the middle of certain segments (e.g. S1, S2, S4, S7). The vPAR-CL signals of RV (Figure 13) by the three smallest segments (S9-S11) contained a modest number of prominent signals while the larger segments such as S1 (25.4 sites per kb), S2 (30.8 sites per kb), S3 (30.9 sites per kb) and S4 (32.6 sites per kb) generally had a higher number, similar to that of BTV data.

## DISCUSSION

The interaction between capsid proteins and viral RNA are essential for the correct genome packaging of many viruses. Lentiviruses, coronaviruses, and influenza viruses selectively package their genomes with the help of their respective nucleoproteins (45–47). Other viruses, such as MS2, BMV and CCMV, use their capsid protein, which also directly recognises the viral RNA via specific sequences or secondary structure motifs (48). Members of the *Reoviridae* lack nucleoproteins and instead have multi-layered capsids, with the inner capsid layer interacting directly with the viral polymerase and very probably with the viral genome which is present as dsRNA. Furthermore, as the dsRNA genome is multi-segmented, it is plausible that the interaction with RNA will may assist in the packaging process to ensure that all of the RNA segments are properly localized within the capsid.

BTV is an attractive model system for segmented dsRNA viruses since the mechanism of replication is well-studied and it has an established system of reverse genetics to confirm the influence of targeted mutations on virus replication. Our previous studies established that VP6 is an RNA packaging protein and that it recognizes the BTV genome in a specific manner (9,18). Furthermore, VP6 binds the VP3 capsid protein, indicating that VP3 may also play a role in RNA binding (8,9,12). In this study, using an RNA capture assay (RCAP), we have demonstrated that VP3 indeed interacts with genomic RNA directly and we have identified at least some specific amino acids where RNA binds. Furthermore, through the use of the RG system we show that this VP3–RNA interaction is essential for virus viability. The inner capsid proteins, the VP1 polymerase and the VP4 capping enzyme, had previously been shown to form a complex within the VP3 capsid layer (17). In addition, recent *in situ* atomic structures have revealed that the interaction between VP1 and the VP3 capsid proteins is crucial for the precise localisation of VP1 and the synthesis of ssRNA transcripts. The RCAP data here show that one of the RNA-interacting regions of VP3 is located in its apical domain in close proximity to VP1 within the core. Further, a substitution mutation in this region failed to package RNA in an *in vitro* RNA packaging assay indicating that this region may also be responsible for RNA packaging. The other RNA-interacting region is located in the carapace domain, near the interface of VP3A and VP3B. Changes in this region perturbed the capsid structure formation, suggesting that this region is important for VP3 structural integrity.

The RCAP analysis of the core also identified three RNA-interacting regions within the VP1 polymerase. The helix bundle of the NTD has been shown by structural analysis to have the potential to interact with RNA termini (36) whilst the palm subdomain contains the essential polymerase active motif GDD (41). The third RNA binding region is in the C-terminal bracelet domain, which is responsible of separation of duplex RNA during transcription (36). Substitution mutations introduced into these regions were found to compromise virus replication consistent with the structural data.

We also applied RCAP analysis to RV, a distinct member of the *Reoviridae* with a similar double-layered core structure (DLPs). RCAP analysis revealed that RV VP2, the equivalent of BTV capsid protein VP3, has a major role in interacting with the encapsidated RNA. One RNA-interacting site located in its apical domain and also one in carapace domain, similar to BTV VP3. Further, it has two additional RNA-interacting sites at its N-terminal domain. The RV VP2 N-terminal domain has been reported previously to interact with the RV RdRp (49). Recent structural analysis revealed that an N-terminal amphipathic helix (aa 75–100) of RV VP2 acts as a transcriptional factor (43), this region also harbours the RNA-interacting region (aa 86–95) identified by our RCAP analysis. Additionally, this region has been shown to undergo a significant conformational change when the capsid shifts from the transcription initiation state (Duplex-open state) to the transcript elongation state (43) consistent with an interaction between VP2 and RNA. We identified one RNA-interacting region (aa 407–431) in the RV RdRp, VP1, located in the finger domain, in close proximity to the short-unwound helix (aa 398–401) which accommodates the incoming template (–) RNA. By analogy, the region encompassing aa 407–431 may be involved in the entry of template genomic RNA (6). It is known that RV RdRp VP1 relies on interactions with capsid protein VP2 for its activities (50–52) unlike the BTV VP1 which could produce dsRNA on (+)ssRNA templates in the absence of VP3 (53). The extra interactions between N-terminal domain of VP2 and RNAs may be necessary for *in vivo* BTV transcription specificity.

In contrast to the VP1 polymerases of both BTV and RV, the *in situ* structures and locations of both the BTV and RV capping enzymes, VP4 and VP3 respectively, have yet to be unambiguously identified. In this study, the RCAP analysis of capsids showed that both BTV and RV capping enzymes bind genomic RNA at the 2’OMTase domain and that the residues concerned were important, as confirmed using site-specific mutagenesis followed by virus recover using RG system for BTV.

Using the vPAR-CL technique with BTV identified capsid protein interacting sequences within the dsRNA virus genome. The distribution of vPAR-CL sites across the 10 genome segments of BTV was not uniform, with the larger segments having more capsid interaction regions per kb and certain smaller segments (S8, S9) having very limited capsid contacts. we speculate that the larger segments are located closer to the inside surface of the capsid and maybe accessed by the transcription complex more easily. It has recently been demonstrated by cryo-EM that the organisation of the genome within dsRNA virus capsids is ordered (7); however, cryo-EM analysis neither identified which RNA segments of the genome are located closer to the capsid nor the sequences that bind to the capsid protein. Among the smaller segments, only S10 harbours a high density of proteins-interacting sites. Previously it has been demonstrated that the S10, which has the longest 3’ UTR among all 10 segments, plays an essential role in BTV genome packaging (23,54).

However, despite the importance of the organisation of the RNA genome within the capsid for RNA packaging and virus assembly, the spatial organization of different dsRNA segments, unlike the non-segmented ssRNA viral genomes (55), remains challenging from available RNA secondary structure mapping methods. The information in the current study might therefore provide vital constraints when performing *in silico* modelling and/or the prediction of the supramolecular organisation of authentic virus particles.

Since viruses have a limited number of capsid proteins that can interact with the genome, it would be expected that viral proteins would recognise RNAs via similar sequences or secondary structures, as in the case of ssRNA viruses (56). STREME analysis identified a potential protein-interacting motif in each RNA segment of BTV, and these were demonstrated to be essential by virus recovery using the RG method. Interestingly, for BTV, the majority of these motifs-containing sites which bound rVP6 *in vitro*, suggesting that these sites might initiate the packaging process with VP6 before relocating to VP3 upon encapsidation. Further study is required to confirm this theory. Although less investigated, RNA-interacting sites for the RV genome for all three DLP capsids proteins was comparable to that of BTV, with larger segments having more capsid interaction sites per kb than the smaller segments suggesting that the mechanism uncovered here may be conserved in both viruses and possibly other members of the *Reoviridae*.

## DATA AVAILABILITY

The sequencing data have been deposited with the NCBI SRA under BioProject ID PRJNA907830.

## Supplementary Material

gkad274_Supplemental_Files
