# Supplementary material for: A multidisciplinary approach to the identification of the protein–RNA connectome in double-stranded RNA virus capsids
Source: Nucleic Acids Res. 2023 Apr 18;51(10):5210–27. doi: 10.1093/nar/gkad274 (PMC10250232; doi:10.1093/nar/gkad274)
Supplement: gkad274_Supplemental_Files [file gkad274_supplemental_files.zip › supplementary figures and tables.pdf]

A

```
MAAQNEQRPERIKTTPYLEGDVLSSDSGPLLSVFALQEIMQKVRQVQADY 50
MTATREVDFTVPDVQKILDDIKALAAEQVYKIVKVPSISFRHIVMQSRDR 100
VLRVDTYYEEMSQVGDVITEDEPEKFYSTIIKKVRFIRGKGSFILHDIPT 150
RDHRGMEVAEPEVLGVEFKNVLPVLTAEHRAMIQNALDGSIIENGNVATR 200
DVDVFIGACSEPIYRIYNRLQGYIEAVQLQELRNSIGWLERLGQRKRITY 250
SQEVLTDFRRQDTIWVLALQLPVNPQVVWDVPRSSIANLIMNIATCLPTG 300
EYIAPNPRISSITLTQRITTTGPFAILTGSTPTAQQLNDVRKIYLALMFP 350
GQIILDLKIDPGERMDPAVRMVAGVVGHLLFTAGGRFTNLTQNMARQLDI 400
ALNDYLLYMYNTRVQVNYGPTGEPLDFQIGRNQYDCNVFRADFATGTGYN 450
GWATIDVEYRDPAPYVHAQRYIRYCGIDSRELINPTTYGIGMTYHCYNEM 500
LRMLVAAGKDSEAAYFRSMLPFHMVRFARINQIINEDLHSVFSLPDDMFN 550
ALLPDLIAGAHQNADPVVLDVSWISLWFAFNRSFEPTHRNEMLEIAPLIE 600
SVYASELSVMKVDMRHLSLMQRRFPDVLIQARPSHFWKAVLNDSPEAVKA 650
VMNLSHSHNFINIRDMMRWVLLPSLQPSLKLVLEEEAWAAANDFEDLMLT 700
DQVYMHRDMLPEPRLDDIERFRQEGFYYTNMLEAPPEIDRVVQYTYEIAR 750
LQANMGQFRAALRRIMDDDDWVRFGGVLRTVRVKFFDARPPDDILQGLPF 800
SYDTNEKGGLSYATIKYATETTIFYLIYNVEFSNTPDSLVLINPTYTMTK 850
VFINKRIVERVRVGQILAVLNRRFVAYKGKMRIMDITQSLKMGTKLAAPT 900
V*
```

B

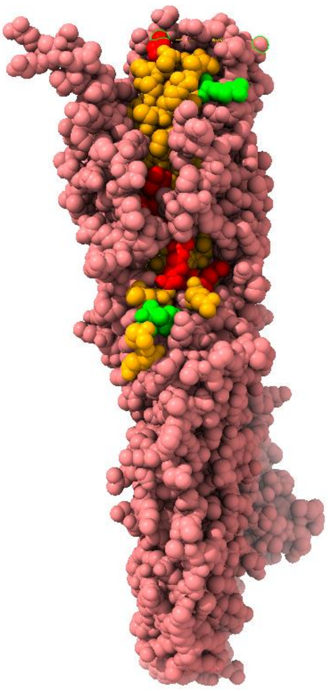

Figure S1. RNA binding regions of BTV-1 VP3. (A) The RCAP identified RNA interacting areas are underlined in red. Positively charged residues are shown in blue. (B) BTV VP3 solved by X-ray crystallography (2btv) are illustrated. RCAP identified interacting regions are coloured orange. Mutated amino acid residues that yield viable virus particles are coloured green, the ones failed to recover virus are coloured red.

A **BTV VP3**

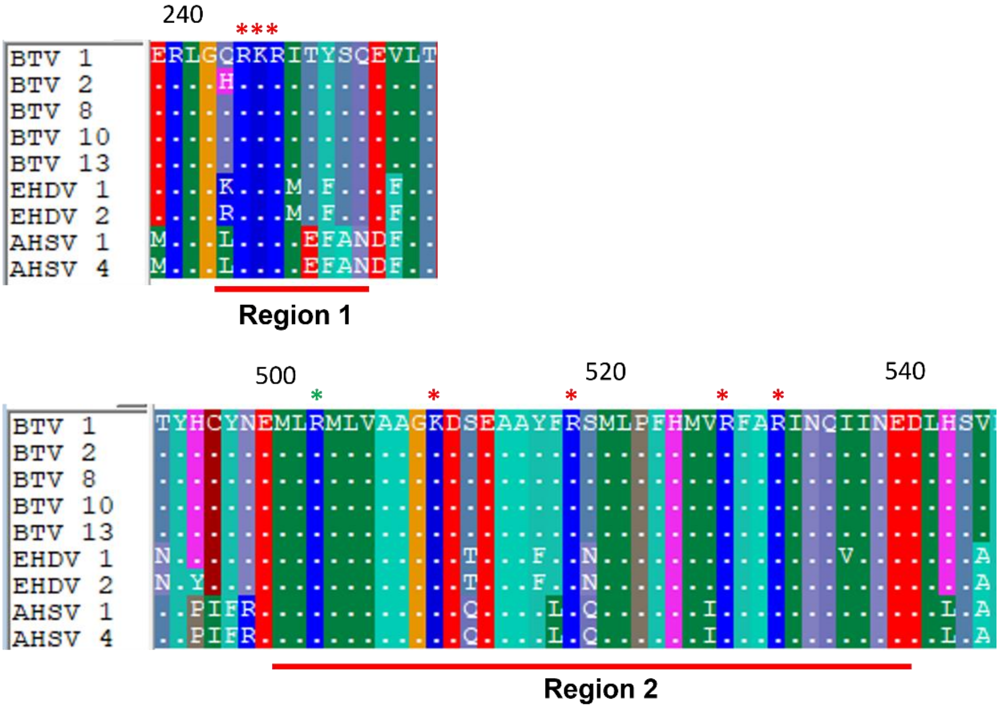

B **BTV VP1**

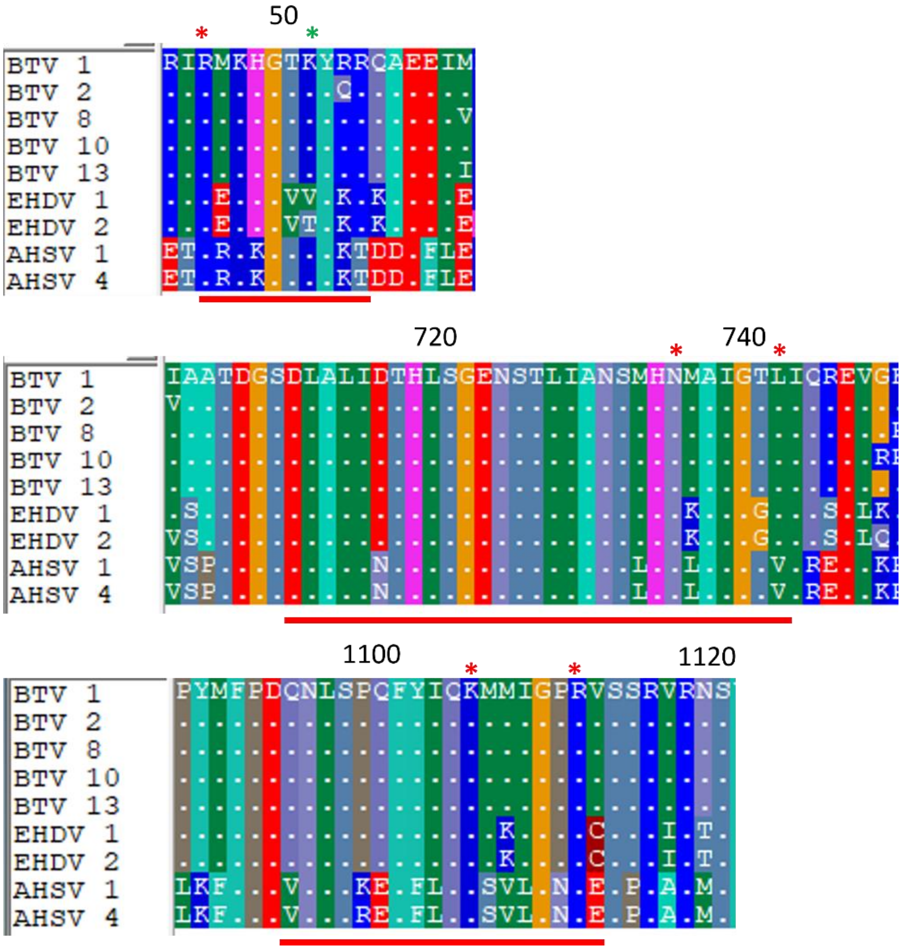

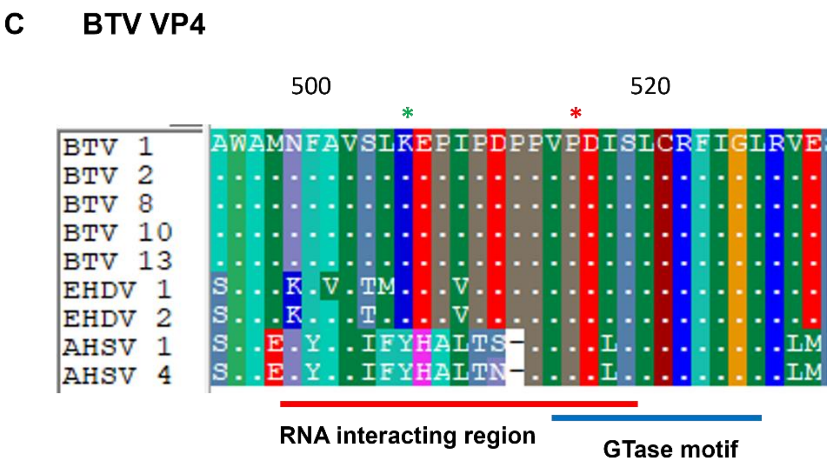

Figure S2. RNA binding sites in VP3, VP1 and VP4 are conserved among BTV serotypes and other Orbiviruses. Alignments of VP3 (A), VP1 (B), and VP4 (C) from different serotypes of BTV (BTV 1: RSA, p56582; BTV 2: IND2010-KRM08; BTV 8: 19-03; BTV 10: isolate 2627; BTV 13: 16.01) and other Orbiviruses (EHDV 1: New Jersey; EHDV 2: Ibaraki; AHSV 1: H29/62; AHSV 4: 91 00) are shown, covering the RNA binding regions (red bars). The GTase motif in VP4 is indicated (blue bar). Residues tested with RG are marked (*), red: not recovered; green: recovered.

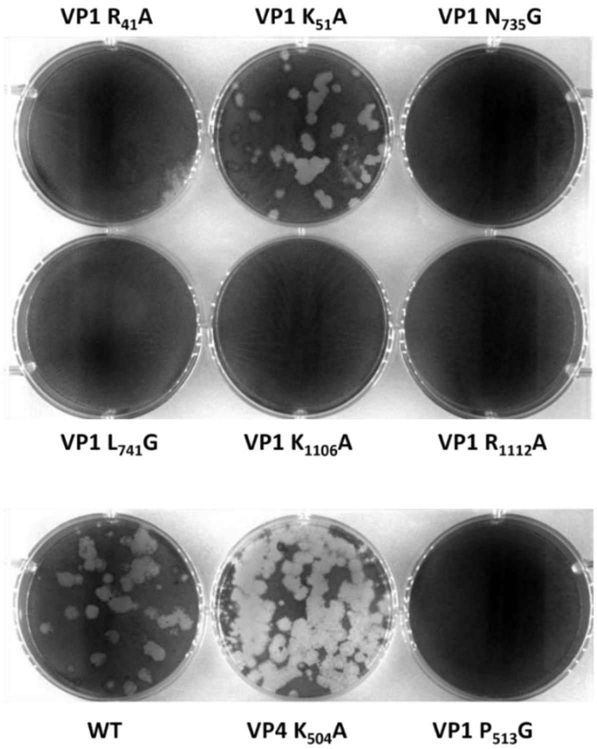

Figure S3. Plagues of BTV VP1 and VP4 mutant viruses. BSR cells monolayers infected with BTV WT and mutants are stained with crystal violet at 48 h post-infection. The VP1 and VP4 mutants are indicated.

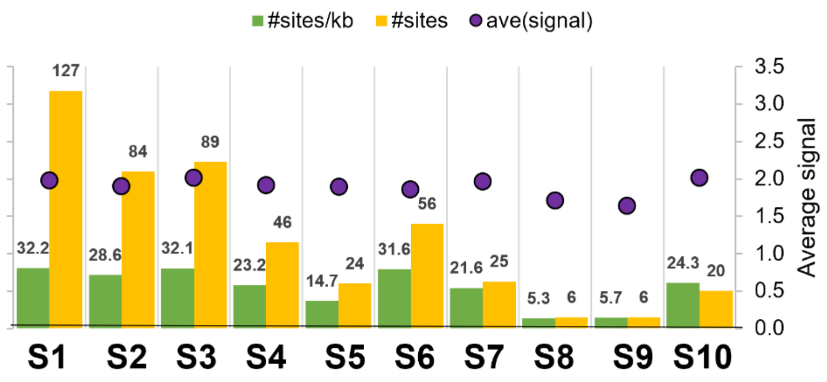

Figure S4. The distribution of protein interacting sites in genomic RNAs / rVP6. The numbers of protein interacting sites (#sites) and ratios of sites per kb (#sites/kb) on each segment are shown.

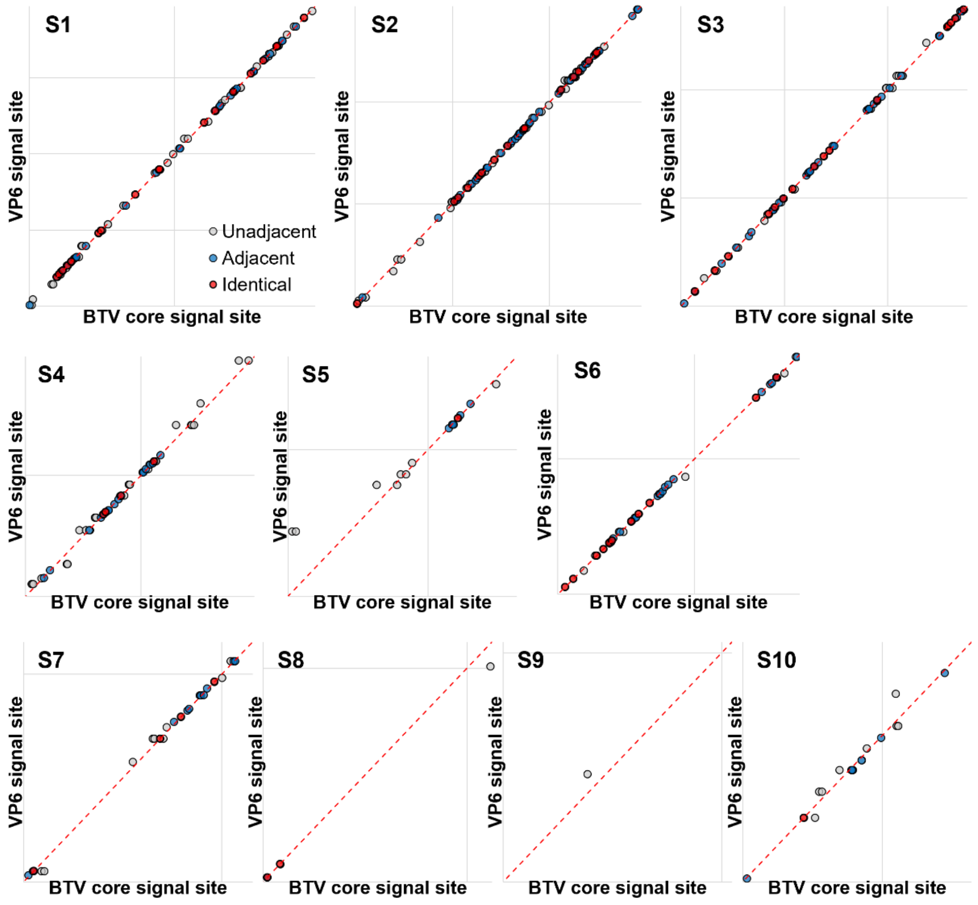

Figure S5. The significant BTV sites (x axis) and their proximity to significant VP6 sites (y axis).

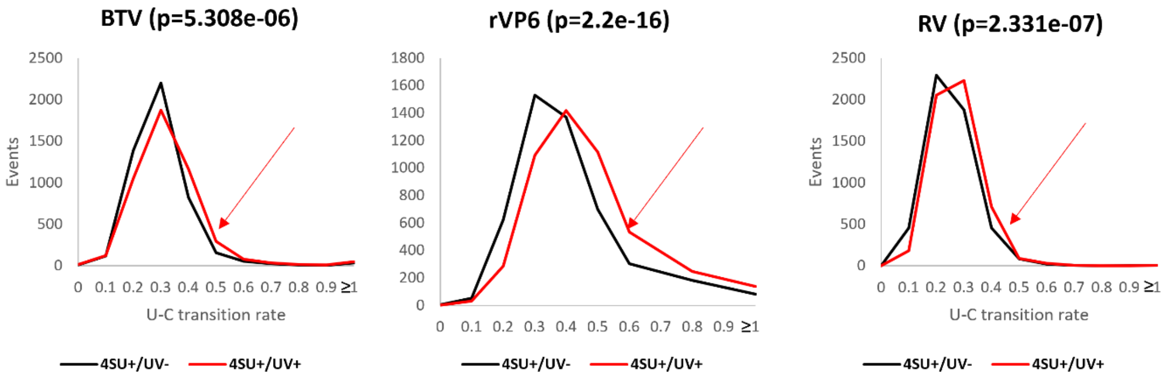

Figure S6. Two-sample Kolmogorov-Smirnov tests for BTV, rVP6 and RV. U-C transition rate increase is specific to crosslinking. Histograms of specific U-C transition rate change of BTV core, rVP6 in vitro binding, and Rota A core. Black line: U-C transition rate of correspondent control group (4SU+/UV-) of each experiment. Red line: U-C transition rate of crosslink group (4SU+/UV+) of each experiment.

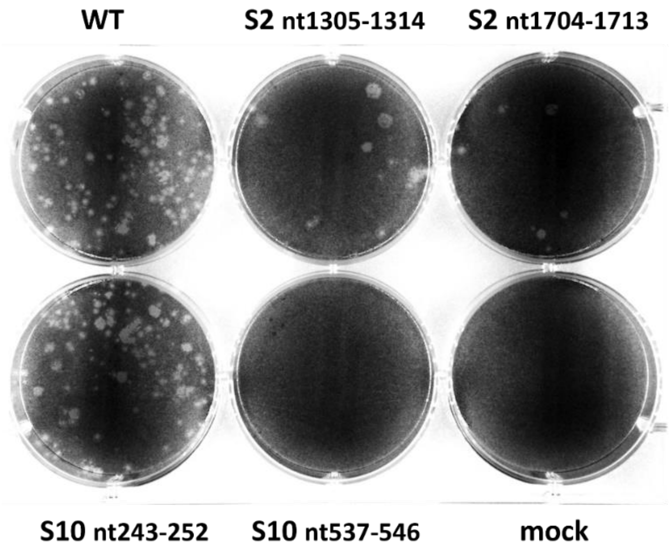

Figure S7. Plagues of BTV mutant viruses with altered S2 and S10 sequences. BSR cells monolayers infected with BTV WT and Mutants are stained with crystal violet at 72h post-infection. The mutants with nucleotides altered are indicated.

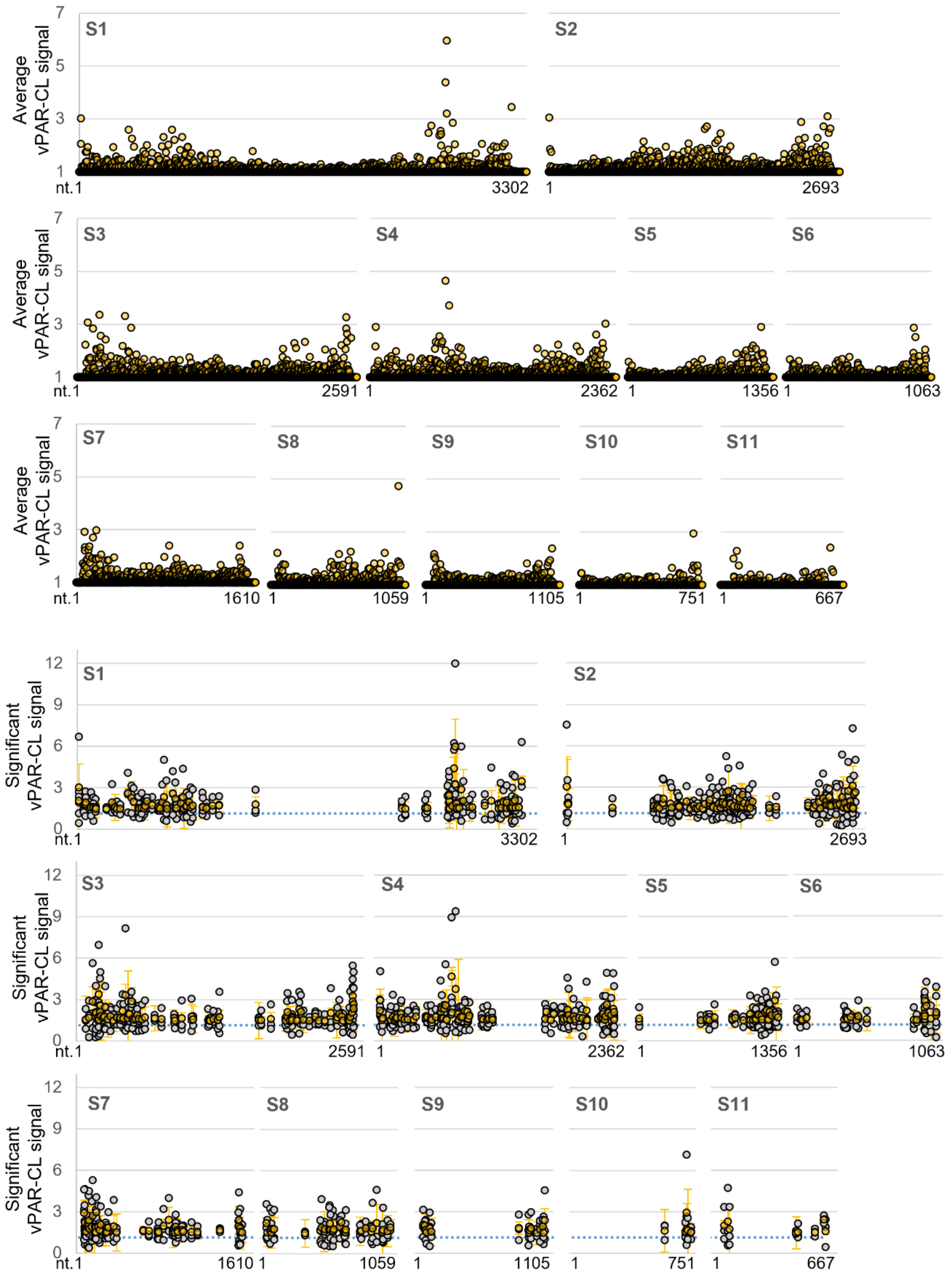

Figure S8. The significant protein interacting sites in RV SA11 genome identified by vPAR-CL.

Table S1. List of primers used for BTV mutagenesis.

**Protein mutation**	**F/R**	**Sequence (5'-3')**
VP1 R45A	F	GCATAGCTATGA AACATGGAACGAAATATCG
	R	GTTTCATAGCTATGCGTCGTATATGATCAGAAAAC
VP1 K51A	F	GGAACGGCTTATCGACGGCAGGCGGAAGAG
	R	GTCGATAAGCCGTTCCATGTTTCATCCTTATG
VP1 N735G	F	GCACGGAATGGCTATTGGGACCTTGATAC
	R	GCCATTCCGTGCATCGAGTTAGC
VP1 L741G	F	GACCGGAATACAACGAGAAGTTG
	R	GTATTCCGGTCCCAATAGCCATGTTGTG
VP1 K1106A	F	CTATATACAGGCTATGATGATTGGCCCAC
	R	CATCATAGCCTGTATATAGAACTGCGG
VP1 R1112A	F	CCCAGCTGTTAGTTCACGAGTGCGGAATTC
	R	CTAACAGCTGGGCCAATCATCATCTTC
VP4 K504A	F	CGTTAGCTGAGCCGATACCTGACCCCCC
	R	GTATCGGCTCAGCTAACGAGACGGCAAAAT
VP4 P513G	F	CTGTGGGAGATATCTCCCTATGTAGATTC
	R	GATATCTCCCACAGGGGGGTCAGGTATCGGC
VP3 RKR 245-7AAA	F	GTTAGGACAGGCTGCTGCTATTACATATTCA CAAGAAGTTC
	R	GAATATGTAATAGCAGCAGCCTGTCCTAA CCTTTCCAACC
VP3 R247A	F	CAGAGGAAAGCTATTACATATTCA CAAGAAGTTC
	R	TATGTAATAGCTTTCCTCTGTCCTAA CCTTTC
VP3 YS 250-1AA	F	GGATTACAGCTGCTCAAGAAGTTCTAACTGACTT TAG
	R	CTTCTTGAGCAGCTGTAATCCTTTTCCTCTGTCC
VP3 S251A	F	TACATATGCTCAAGAAGTTCTAACTGAC
	R	CTTCTTGAGCATATGTAATCCTTTTCCTCTGTCC
VP3 K509A	F	GCTGCAGGGGCTGATTCTGAAGCGGCGT AC
	R	CAGAATCAGCCCCTGCAGCAACTCG
VP3 R526A	F	CATATGGTAGCTTTCGCTAGAATAAAC CAAATC

	R	CTAGCGAAAGCTACCATATGAAAGGGTAAC
R529A	F	GTTCGCTGCTATAAACCAAATCATAA ATG
	R	GGTTTATAGCAGCGAACCTTACCATATG

**Segment**	**Start of motif**	**F/R**	**Sequence (5' – 3')**
S1	523	F	CGTGACCTGAG CAACCTAGAA GATTTTCAAG TTGCATACAC
		R	CTTCTAGGTTGCTCAGGTCACGATTCCCCTGATGGAATG
S2	1305	F	GA ATACCGTTTA AACCATTCCA CCCGGGAGAT AACCTATG
		R	GAATGGTTTAAACGGTATTCCCAATGAACAATTTTAATC
S2	1704	F	CAACGACTCA CACTGGCGCG GTTTTACGAC ATTCGTCC
		R	GCCAGTGTGAGTCGTTGCAATTCGATTGGATCTGCGG
S3	1828	F	TATGCAA GGGATCTTTC CGTGATGAAG GTAGATATGC
		R	CGGAAAGATCCCTTGCATAAACGGATTCGATCAGCGG
S3	2617	F	GATTTTAGC AGTGCTAAAC AGAAGATTTG TGGCATATAA AG
		R	CTGTTTAGCACTGCTAAAATCTGTCCAACCCGTACTCG
S4	601	F	GGATGTGGAGATCT TCGTACCTTG ATGCAGTTTA
		R	**GGTACGAAGATCTCCACATCCAACGTAGTGCACCTCC**
S10	243	F	GAGAAAGCAGCT TTTGCATCGT ACGCAGAAGC GTTTCGTG
		R	GATGCAAAAGCTGCTTTCTCCGCTTTTTGTGTTTGCGTAGC
S10	537	F	GGGAGC AACATTTTTA ATGATGGTAT GCGCAAAAAG CGAG
		R	CATACCATCATTAAAAATGTTGCTCCCAAATTCACCACACCTAAC

Table S2. List of BTV peptides identified in BTV cores RCAP.

	Protein	# ms/ms	Sequence	Starting AA #	Modifications	m/z [ppm]	m/z Error [ppm]	Confidence (%)
**Crosslinked Cores**	VP1	1	TTPLLFEMCCMESILEFNIKMRMREE	200	Oxidation of M(8, 11)	1076.2	-4.6	58
	VP1	1	NRYKALFR	305		534.8	0.5	38
	VP1	2	IYVKKRFGPRLRDKDLIK	456		562.4	-2.2	100
	VP1	1	QKALNSWIAQVSMRLGE	1049		483.8	-4.1	61
	VP1	1	IQKMMIGPRV	1104		586.8	-0.6	73
	VP1	1	VIEKLGTNHSVGDLVTVFT	1147		1015.6	7.5	63
	VP1	1	DEFTMSLNVATQDFI	1201		865.9	-0.7	81
	VP3	1	VKVPSISFRHIVMQSRDRVLRV	83	Oxidation of M(13)	660.38		100
	VP3	1	EPEKFYSTIIKKV	122		528.31	3.1	100
	VP3	1	QRKRITYSQE	244		655.4	-1.4	88
	VP3	1	RMDPAVRMVAGVVGHLLFTAG	364		1115.1	-5	75
	VP3	1	HCYNEMLRMLVAAGKDSEAAYFRS	495	Oxidation of M(9)	926.77	0.9	100
	VP3	1	MLRMLVAAGKDS	500	Oxidation of M(4)	654.3	-0.4	100
	VP3	1	LRMLVAAGKDS	501	Oxidation of M(3)	589.3	-3.4	84
	VP3	1	SMLPFHMVRFARINQIINE	518	Oxidation of M(7)	778.4	-2.3	91
	VP3	1	RFARINQIIN	526	Oxidation of M(4)	564.3	-0.6	84
	VP4	2	GFGDALRN	116		425.21	-0.3	78
	VP4	1	EIPPLYMEYAEIGTRFDDEPTDEKLVSMLDY	155	Oxidation of M(28,7)	1232.24	0.5	67
	VP4	1	CISTYLLPQPGADADM	318	Oxidation of M(16)	856.39	2.9	68
	VP6	3	WVVLTEEIAR	132		608.34	0.9	100
	VP6	21	IDVYRDEVPAQIIEVER	151		682.03	-0.22	100
	VP6	7	DEVPAQIIEVER	156		699.37	0.35	100
	VP6	1	EGTEEEKTSEEPASVGITIEGVMSQK	221		928.11	1.8	100
	VP6	5	KLLSMIGGVER	247		407.23	-0.1	100
	VP6	1	KLLSMIGGVER	247	Oxidation of M(23)	609.85	0.8	100
	VP6	12	KLLSMIGGVER	247	Oxidation of M(5)	406.9	0.84	100
	VP6	6	LLSMIGGVER	248	Oxidation of M(5)	545.8	-1.8	100
	VP6	8	LLSMIGGVER	248	Oxidation of M(4)	545.8	1.2	100
	VP6	7	ESAVMLVSNSIK	266	Oxidation of M(4 or 5)	647.34	-1.1	100
	VP6	10	ESAVMLVSNSIKDVVR	266	Oxidation of M(4 or 5)	881.97	0.6	100
	VP6	1	VSNSIKDVVR	272	Oxidation of M(5)	558.82	2.9	94

	VP6	5	ATAYFTAPTGDPHWK	282	Oxidation of M(5)	554.93	-1.4	100
	VP6	17	ATAYFTAPTGDPHWK	282		831.9	-1.1	100
	VP6	2	KNILAYTSTGGDVK	306		733.9	0.6	99
	VP6	16	NILAYTSTGGDVK	307		669.85	-3.7	100
	VP6	4	NILAYTSTGGDVKTE	307		784.89	1.45	100
	VP6	13	NILAYTSTGGDVKTEFLHLIDHL	307		853.12	3.2	94
	VP6	21	TEFLHLIDHL	320		413.22	1.2	100
**Crosslinked, no RNA**	VP4	1	KEPIPDPPVPDI	504			-1.3	77
	VP6	1	WVVLTEEIAR	132		608.34	2.2	100
	VP6	1	IDVYRDEVPAQIIEVER	151		682.03	1.9	100
	VP6	1	IDVYRDEVPAQIIEVERSLQKELGISR	151		789.68	2.9	92
	VP6	1	DEVPAQIIEVER	156		699.37	-0.1	100
	VP6	2	KLLSMIGGVER	247	Oxidation of M(5)	406.9	0.0	90
	VP6	1	LLSMIGGVER	248	Oxidation of M(4)	545.8	1.9	100
	VP6	1	LLSMIGGVERK	248	Oxidation of M(4)	609.85	-2.6	83
	VP6	1	ESAVMLVSNSIK	266	Oxidation of M(5)	647.34	1.1	99
	VP6	1	ESAVMLVSNSIKDVVR	266	Oxidation of M(5)	881.97	0.7	72
	VP6	1	ATAYFTAPTGDPHWK	282		831.9	0.4	99
	VP6	1	KNILAYTSTGGDVKTEFLHLIDHL	306		896.15	2.4	72
	VP6	3	NILAYTSTGGDVK	307		733.9	1.0	100
	VP6	1	NILAYTSTGGDVKTEFLHLIDHL	307		640.09	2.1	80
	VP6	3	TEFLHLIDHL	320		413.22	1.3	100
	VP7	14	IVLEANVMEILGIAINR	23	Oxidation of M(8)	942.54	-3.2	100
**No Crosslinking**	VP1	1	RMKHGTKYRR	45		666.87	-3.2	68
	VP4	1	KEPIPDPPVPDI	504		659.36	-1.3	77
	VP6	1	WVVLTEEIAR	132		608.34	1.0	100
	VP6	1	VVLTEEIAR	133		515.3	3.6	94
	VP6	1	IDVYRDEVPAQIIEVER	151		1022.54	1.6	100
	VP6	1	DEVPAQIIEVER	156		699.37	2.9	100
	VP6	1	KLLSMIGGVER	247	Oxidation of M(5)	406.9	1.3	100
	VP6	1	LLSMIGGVER	248	Oxidation of M(4)	545.8	1.0	96
	VP6	1	ESAVMLVSNSIK	266	Oxidation of M(5)	647.3	0.9	98
	VP6	2	ESAVMLVSNSIKDVVR	266	Oxidation of M(5)	881.97	1.0	98

	VP6	1	NSIKDVVR	274		465.77	4.7	81
	VP6	2	ATAYFTAPTGDPHWK	282		554.93	1.1	100
	VP6	1	ATAYFTAPTGDPHWKEVAR	282		706.69	0.1	79
	VP6	1	KKNILAYTSTGGDVK	305		733.9	-2.4	98
	VP6	1	KNILAYTSTGGDVK	306		489.6	2.0	92
	VP6	1	KNILAYTSTGGDVKTEFLHLIDHL	306		896.15	0.3	86
	VP6	3	NILAYTSTGGDVK	307		733.9	2.9	100
	VP6	1	NILAYTSTGGDVKTE	307		489.6	-0.5	82
	VP6	2	NILAYTSTGGDVKTEFLHLIDHL	307		640.09	2.5	90
	VP6	1	TEFLHLIDHL	320		413.22	1.4	100
	VP7	18	IVLEANVMEILGIAINR	23	Oxidation of M(8)	942.54	-1.2	97

Table S3. List of RV SA11 peptides identified in RV DLPs RCAP.

	Protein	# ms/ms	Sequence	Starting AA #	Modif.	m/z (ppm)	m/z error (ppm)	Confidence (%)
**Crosslinked**	VP1	1	QLKTEYTEDVDGEMYNEYTMLIRDEIVKM	346	Oxidation of M(20, 14)	897.9	4.5	100
	VP1	1	KFGRKTIFSTKKNMHVMDDIAHG	409	Oxidation of M(14)	893.1	-1.9	100
	VP1	1	IIPPVNVDKPIPL	437		708.4	1.6	89
	VP1	1	KLVNYAKG	88		446.8	3.3	87
	VP1	1	QFNTDVTKQMVQ	638		719.9	4.8	82
	VP1	1	EMYREYTMLIRDEVVKMLEE	358	Oxidation of M(8, 17, 2)	1313.1	-3.1	83
	VP1	1	IQDWLVDRSI	307		623.3	3.9	79
	VP1	1	QLLISKNVIV	818	Pyrolidone from Q(1)	555.4	6	74
	VP2	22	KEEIITDNQEEVK	46		787.9	3.1	100
	VP2	2	EEIITDNQEEVK	47		723.9	4.8	82
	VP2	8	EVQYEILQK	93		575.3	1.7	100
	VP2	1	TIPTFEPK	102		466.8	3.1	83
	VP2	10	KLEDIKPEQAK	109		433.6	4.2	100
	VP2	1	LEDIKPEQAK	110		585.8	3	100
	VP2	1	MSLDFVTTNYMSLISG	402	Oxidation of M(11)	897.9	-3.2	67

	VP2	1	GMWLLTVIPNDMFIRESLVAC	409	Oxidation of M(12, 2)	814.1	-2.5	100
	VP2	1	LRESLVACELAIINTIVYPAFGMQRMHYR	429	Oxidation of M(23)	1137.6	-6.8	95
	VP2	1	LMNMEQIERASDKIAQGVIIAY	695	Oxidation of M(2)	1255.6	-0.7	92
	VP2	1	ERASDKIAQGVI	702	Pyrolidone from E(1)	423.9	-4.3	68
	VP3	1	ALDFQNFMLKKIKER	162		941.5	-3.3	65
	VP3	1	NFMLKKIKERMTTSLPIARLSNRVF	167	Oxidation of M(3, 11)	1009.6	-3.6	78
	VP3	1	MTTSLPIARLSNR	177	Oxidation of M(1)	738.4	-2.7	100
	VP3	1	YDVRHYSDFFFETWDPLDTPYSSIHHKE	294		883.9	-3	100
	VP6	3	NGVAPQSEALR	107		571.3	2.7	100
	VP6	2	NGVAPQSEALRK	107		635.4	1.82	100
	VP6	2	ALTTATITLLPDAER	217		793.4	-2.4	96
	VP6	3	VFTVASIR	385		446.8	3.45	100
	NSP1	1	RPSPPTKYKGWCLDCCQHTDLTYCQGCTMY	31	Oxidation of M(29)	879.6	-5.2	100
	NSP1	1	PPTKYKGWCLDCCQHTDLTYCQGCTMYHD	34	Oxidation of M(26)	857.6	5.6	100
	NSP1	1	CCQHTDLTYCQGCTMYHDCQWC	45		661.5	-5.9	100
	NSP1	1	VWNDFRVKKIYDNIFNFL	332		1166.6	-0.9	91
	NSP5	1	NDPLTSFS	68		441.2	4.9	82
**No crosslinking**	VP2	4	KLEDIKPEQAK	109		433.6	5.9	100
	VP2	9	KEEIITDNQEEVK	46		787.9	0.04	100
	VP2	1	YNYETLMACITMNMQHVQT	561	Oxidation of M(7)	1154.5	4.5	95
	VP3	1	YDVKVPHLTLGVLFISDNMI	712	Oxidation of M(19)	1145.6	-6.6	79
	VP6	2	NGVAPQSEALRK	107		635.4	0.3	100
	VP6	1	ERFSFPRVINSADGATTWFFNP	230		854.1	-2.9	76
	VP6	1	VFTVASIR	385		446.8	3.5	100
	NSP2	1	VLSRVVSVRHLENLVLRKE	101		562.4	7.7	100
	NSP3	1	LIRNLIQDYDRTFLMLKGLLKQCNYE	284	Oxidation of M(15)	1073.2	4.3	63.6
